# Effects of glycogen synthase kinase-3β activity inhibition on cognitive, behavioral, and hippocampal ultrastructural deficits in adulthood associated with adolescent methamphetamine exposure

**DOI:** 10.3389/fnmol.2023.1129553

**Published:** 2023-03-06

**Authors:** Peng Yan, Jincen Liu, Haotian Ma, Yue Feng, Jingjing Cui, Yuying Bai, Xin Huang, Yongsheng Zhu, Shuguang Wei, Jianghua Lai

**Affiliations:** ^1^NHC Key Laboratory of Forensic Science, School of Forensic Sciences, Xi’an Jiaotong University, Xi’an, China; ^2^Forensic Identification Institute, The Fourth People’s Hospital of Yancheng, Yancheng, China

**Keywords:** methamphetamine, adolescence, glycogen synthase kinase-3β (GSK3β), CA1 – Cornu ammonis region 1, recognition memory, hyperactivity

## Abstract

**Objective:**

Glycogen synthase kinase-3β (GSK3β) has been implicated in the maintenance of synaptic plasticity, memory process, and psychostimulant-induced behavioral effects. Hyperactive GSK3β in the Cornu Ammonis 1 (CA1) subregion of the dorsal hippocampus (DHP) was associated with adolescent methamphetamine (METH) exposure-induced behavioral and cognitive deficits in adulthood. This study aimed to evaluate the possible therapeutic effects of GSK3β inhibition in adulthood on adolescent METH exposure-induced long-term neurobiological deficits.

**Methods:**

Adolescent male mice were treated with METH from postnatal day (PND) 45–51. In adulthood, three intervention protocols (acute lithium chloride systemic administration, chronic lithium chloride systemic administration, and chronic SB216763 administration within CA1) were used for GSK3β activity inhibition. The effect of GSK3β intervention on cognition, behavior, and GSK3β activity and synaptic ultrastructure in the DHP CA1 subregion were detected in adulthood.

**Results:**

In adulthood, all three interventions reduced adolescent METH exposure-induced hyperactivity (PND97), while only chronic systemic and chronic within CA1 administration ameliorated the induced impairments in spatial (PND99), social (PND101) and object (PND103) recognition memory. In addition, although three interventions reversed the aberrant GSK3β activity in the DHP CA1 subregion (PND104), only chronic systemic and chronic within CA1 administration rescued adolescent METH exposure-induced synaptic ultrastructure changes in the DHP CA1 subregion (PND104) in adulthood.

**Conclusion:**

Rescuing synaptic ultrastructural abnormalities in the dHIP CA1 subregion by chronic administration of a GSK3β inhibitor may be a suitable therapeutic strategy for the treatment of behavioral and cognitive deficits in adulthood associated with adolescent METH abuse.

## Introduction

Methamphetamine (METH) is a highly addictive psychoactive substance, and its abuse has become an important global public health concern (UNODC, 2019). Adolescence is a special period of susceptibility to drug abuse, and METH use is often initiated during this period (Ye et al., 2014). Although a large number of METH users are adults, the number of adolescent METH users has increased rapidly over the past decade (Nazari et al., 2020; Basedow et al., 2021). Adolescence is also a substantial period of brain development, making this an especially vulnerable period for neurotoxic damage (Luikinga et al., 2018). METH exposure in adolescence can cause long-lasting effects on the developing brain, resulting in a series of abnormalities in behavior, cognition, and brain structures in adulthood, even after prolonged periods of drug abstinence (Spear, 2016). Thus, studying how to improve adolescent METH exposure-induced long-term neurobiological deficits in adulthood is necessary.

One key reason why adolescent METH exposure induces long-lasting impairments is that METH may disorganize the normal pattern of growth and maturation of the brain (Westbrook et al., 2020). The hippocampus is one of the important regions of the limbic system, undergoing significant restructuring and maturation in adolescence (Fuhrmann et al., 2015; Lee and Kim, 2019). The hippocampus not only plays a crucial role in learning, memory, and locomotion but is also a target for psychostimulants (Tanimizu et al., 2017; Shukla and Vincent, 2021). Adolescent METH exposure induces profound impairments in reference memory and spatial memory, decreases hippocampal plasticity, and leads to hippocampal cell damage in adulthood (Vorhees et al., 2005; North et al., 2013; García-Cabrerizo et al., 2018). In addition, a previous study showed that adult mice with a history of METH administration in adolescence exhibited significant alterations in locomotor activity, novel spatial exploration, and social recognition memory, as well as abnormalities in excitatory synapse density and postsynaptic density (PSD) thickness in the Cornu Ammonis 1 (CA1) subregion of the dorsal hippocampus (DHP; Yan et al., 2019). These results highlight that the hippocampus is sensitive to adolescent METH exposure-induced long-term nerve damage in adulthood, indicating that recovery of hippocampal function may be a treatment strategy for behavioral and cognitive deficits in adulthood associated with adolescent METH abuse.

Glycogen synthase kinase-3β (GSK3β) is a multifunctional Ser/Thr kinase that is highly expressed in the hippocampus, prefrontal cortex, and other brain regions (Demuro et al., 2021). As a regulator of several cellular processes, GSK3β has a central position in the control of emotion, locomotion, and memory (Chen et al., 2021; Jung et al., 2021). An increasing number of studies have indicated an important role of GSK3β in the effects of psychostimulants (Barr and Unterwald, 2020). METH exposure prominently modulates GSK3β activity, whereas inhibition of GSK3β activity can ameliorate METH exposure-induced hyperactivity, locomotor sensitization, and neurotoxicity (Xu et al., 2011; Wu et al., 2015; Xing et al., 2015). Moreover, adolescent METH exposure significantly enhanced GSK3β activity in both the medial prefrontal cortex (mPFC) and DHP by regulating the phosphorylation pattern of GSK3β, but after prolonged METH abstinence, in adulthood, the increased GSK3β activity remained only in the DHP instead of the mPFC (Yan et al., 2019). Thus, METH exposure during adolescence induced long-term dysregulation of GSK3β activity in DHP, which may be a key factor in that induced deficit in cognition and behavior in adulthood. Dysfunction of GSK3β is involved in the pathogenesis of several psychoneuroses; therefore, GSK3β has been considered a therapeutic target for Alzheimer’s disease and bipolar disorder (Bhat et al., 2018; Ochoa, 2022). However, it is unclear whether recovering GSK3β activity in the DHP in adulthood is beneficial to adolescent METH exposure-induced long-term deficits.

The activity of GSK3β depends on site-specific phosphorylation, phosphorylation of Tyr216 (Y216) on GSK3β activates GSK3β, while phosphorylation of Ser9 (S9) on GSK3β inhibits its activity. The role of Ser9 phosphorylation may be much bigger than the role of Tyr216 phosphorylation in GSK3β activity regulation (Takahashi-Yanaga et al., 2004), and Ser9 phosphorylation is the most frequently suggested mechanism regulating GSK3β activity (Beurel et al., 2015). Lithium (Li) is the first GSK3β inhibitor to be identified and widely used in prescription medicine for bipolar disorder treatment (King et al., 2014), and SB216763 has been widely used in GSK3β-related studies and has been found to improve memory impairment, stimulants-induced hyperactivity, behavioral sensitization, and synaptic transmission dysfunction (Xu et al., 2011; Zhao et al., 2016; Lin et al., 2018). Both agents can inhibit GSK3β activity by increasing Ser9 phosphorylation (Xu et al., 2011; Demuro et al., 2021). In the present study, we performed three GSK3β intervention protocols by using Li chloride (LiCl) and SB216763, and aimed to investigate the possible therapeutic effects of GSK3β activity inhibition in adulthood on adolescent METH exposure-induced long-term alterations in behavior, cognition, and hippocampal synaptic plasticity.

## Materials and methods

### Study design

**Figure 1:**
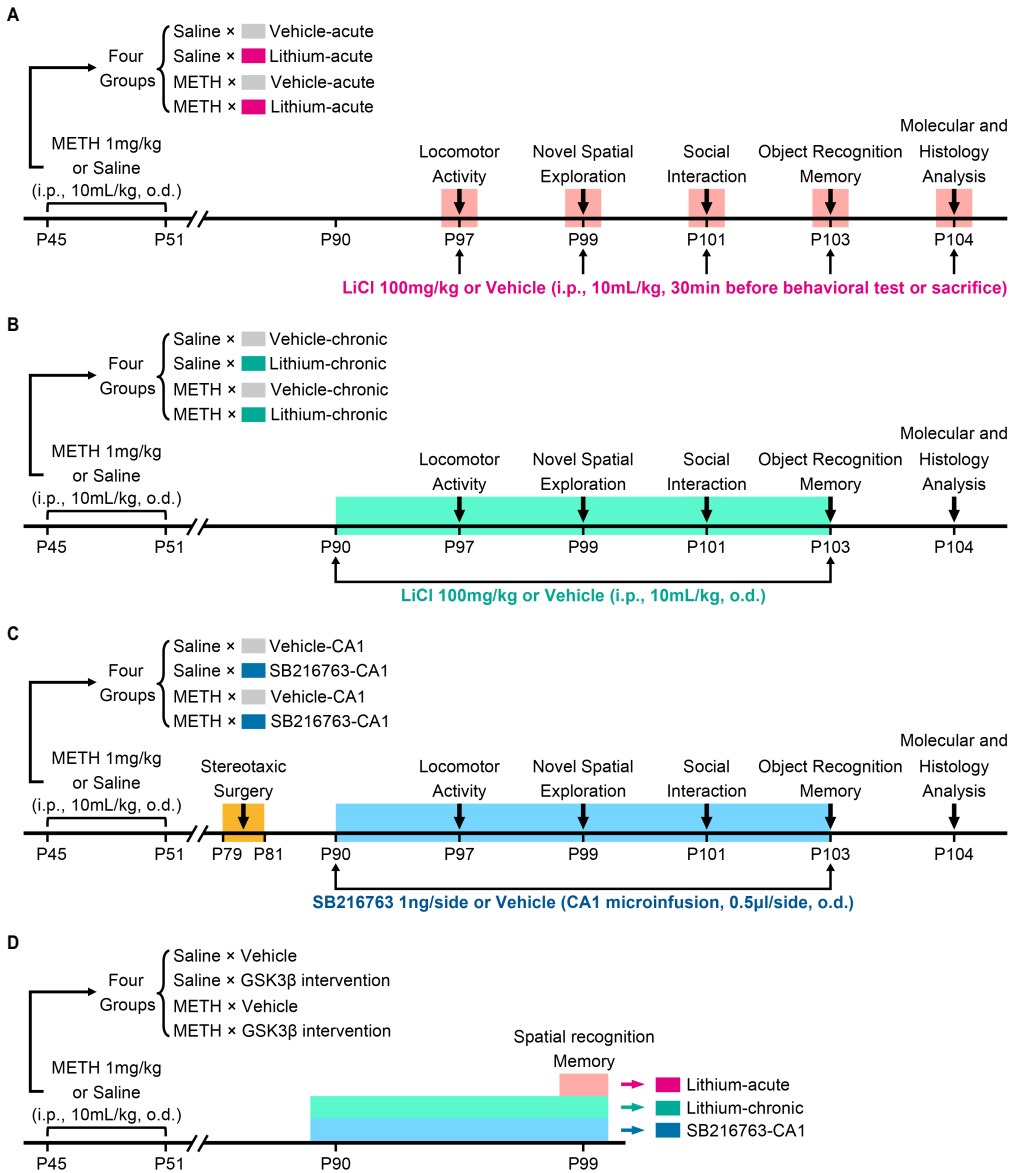
Schematic diagram of GSK3β intervention protocols and experimental time courses. Acute systemic intervention **(A)**, chronic systemic intervention **(B)**, and chronic intervention within CA1 **(C)** were performed to detect the therapeutic effects of GSK3β inhibition on adolescent METH exposure-induced cognitive, behavioral, and hippocampal ultrastructural deficits in adulthood. To further explain the results of novel spatial exploration, spatial recognition memory was detected in a separate cohort of mice **(D)**.

To assess the therapeutic effects of GSK3β inhibition on cognition, behavior, and hippocampal ultrastructural deficits in adulthood associated with adolescent METH exposure, three intervention protocols were used, as schematically shown in Figure 1. In each protocol, mice received the same daily (o.d.) i.p. injection of METH (1 mg/kg) or saline (similar volume to METH) in late adolescence for 7 days from PND 45 to 51 and participated in the same behavioral tests (Brust et al., 2015; Spear, 2015). In our previous study, we found adult mice with a history of METH administration in adolescence exhibited impairments in locomotor activity, novel spatial exploration behavior and social recognition memory, instead of working memory and anxiety-and depressive-like behaviors (Yan et al., 2019). According to this, in the present study, the behavioral tests were selected and performed in the following sequence: an open field test (OFT) for detecting locomotor activity (PND 97), a modified two-trial Y-maze test for detecting novel spatial exploration behavior (PND 99), a three-chamber social behavior test for detecting sociability and social recognition memory (PND 101), and a novel object recognition (NOR) test for further detection of recognition memory (PND 103). Moreover, a standard two-trial Y-maze test was performed in a separate cohort to explain the results of the modified two-trial Y-maze test and to detect spatial recognition memory (PND 99). An overview of the timing of the behavioral tests is provided in Figure 1.

In the acute systemic intervention protocol (Li–acute) (Figure 1A), the mice were randomly divided into the following groups: saline × vehicle–acute, saline × Li–acute, METH × vehicle–acute, and METH × Li–acute. In each group, LiCl (100 mg/kg, i.p.) or saline (similar volume to LiCl, i.p.) was injected 30 min before the behavioral tests and sacrifice.

In the chronic systemic intervention protocol (Li–chronic) (Figure 1B), mice were randomly divided into the following groups: saline × vehicle–chronic, saline × Li–chronic, METH × vehicle–chronic, and METH × Li–chronic. LiCl (100 mg/kg, i.p., o.d.) or saline (similar volume to LiCl, i.p., o.d.) injection was carried out at 18:00 on each day from PND 90 to PND 103.

The therapeutic potential of GSK3β is highly dependent on the brain region. Thus, in the chronic CA1 intervention protocol (SB–CA1) (Figure 1C), we investigated the effect of GSK3β inhibition within the CA1 subregion of the DHP. Mice were randomly divided into the following groups: saline × vehicle–CA1, saline × SB216763–CA1, METH × vehicle–CA1, and METH × SB216763–CA1. SB216763 (1 ng/side, CA1 infusion, o.d.) or vehicle (similar volume to SB216763, CA1 infusion, o.d.) injection was carried out at 18:00 on each day from PND 90 to PND 103.

### Animals

All adolescent male C57BL/6 J mice were obtained at PND 35 and were housed in pathogen-free rooms in groups of four under controlled conditions (12-h light/dark cycle, 50 ± 5% humidity, and 22 ± 3°C temperature control) with food and water *ad libitum*. All mice were acclimated to the environment for 7 days and handled daily for 4 days before the experiment. All animal procedures were approved by the Institutional Animal Care and Use Committee of Xi’an Jiaotong University.

### Drug preparation and administration

Methamphetamine hydrochloride (The Third Research Institute of The Ministry of Public Security, Shanghai, China) and LiCl (Sigma, St. Louis, MO, United States) were dissolved in 0.9% saline to final concentrations of 0.1 and 10 mg/mL, respectively. SB216763 (Sigma, St. Louis, MO, United States) was dissolved in 3% (vol/vol) DMSO, 3% (vol/vol) Tween 80, and distilled water (3:3:94) to a final concentration of 2 ng/μL. All drugs were freshly prepared before use and i.p. injected at a volume of 10 ml/kg or microinfused into CA1 at a volume of 0.5 μL/side.

For CA1 microinfusions, stereotaxic surgery was performed on PND 79–81 to prevent the effects of surgery on brain development. The mice were anesthetized with sodium pentobarbital (65 mg/kg, i.p.) and fixed in a stereotaxic frame. Stainless steel guide cannulae (27G, RWD Life Science, Shenzhen, China) were bilaterally implanted into the CA1 subregion of the DHP at the following stereotaxic coordinates: AP −2.00 mm, ML ± 1.50 mm, and DV −1.00 mm (Gyu et al., 2012). The guide cannulae were secured to the skull using dental cement, and dummy cannulae were inserted. The mice were allowed to recover for 1 week after surgery. For intracranial injection, the mice were restrained carefully, and the dummy cannulae were replaced by internal cannulae (0.5 mm longer than the guide cannulae). SB216763 or the vehicle was bilaterally infused into CA1 at a rate of 0.1 μL/min, and the internal cannula remained in the guide cannula for 5 min after the infusion to ensure the proper delivery of the reagents.

The drug doses used in the present study were chosen based on previous reports and can produce significant biological effects (Kamei et al., 2006; Xu et al., 2011; Gyu et al., 2012; Yan et al., 2019).

### Behavioral tests

All behavioral tests were performed from 8:00 to 16:00 on each test day. Tests were recorded and analyzed using the Any-maze 5.2 software (Stoelting Co., Wood Dale, IL, United States). An entry was defined as all four paws in one area. The apparatus was cleaned using 50% ethanol for different trials and phases. The distal cues consisted of different geometric shapes (including rectangle, circle, triangle, pentagon, and irregular polygon), and were changed between different types of behavioral tests. In all behavioral tests, a white LED light with diffuser plate was suspended 2.5 meters above the center of the apparatus, and the illumination of the apparatus floor was approximately 50 Lux. Previous study reported this illumination may have neither anxiogenic stimulus nor anxiolytic stimulus (Neuwirth et al., 2022).

#### Open field test (locomotor activity)

Mice were individually placed in a plastic box (45 × 45 × 30 cm) and allowed to freely explore the arena for 60 min. The center area (30 × 30 cm) and four corner areas (7.5 × 7.5 cm) were marked using Any-maze 5.2 software. The distance moved, movement duration, and movement in the center and at the four corners were measured.

#### Modified two-trial Y-maze test (novel spatial exploration)

This test was performed as described in previous study (Yan et al., 2019). We used a Y-maze in which the wall of one of the three arms was marked with a black-and-white stripe and defined as the novel arm. Each arm of the Y-maze was 30 cm in length, 6 cm in width, and 15 cm in height. The apparatus was rotated by +60° between tests. First, the novel arm was blocked, and the mice were allowed to habituate to the Y-maze for 5 min. After 30 min of rest, the novel arm was opened, and the mice were allowed to freely explore all three arms for 5 min. The time spent in the novel arm (%) was defined as the time spent in the novel arm divided by the time spent in all three arms. Entries into the novel arm (%) were defined as the number of entries into the novel arm divided by the total number of entries into all three arms. The latency to the first entry and the longest single visit to the novel arm were also recorded.

**Figure 2:**
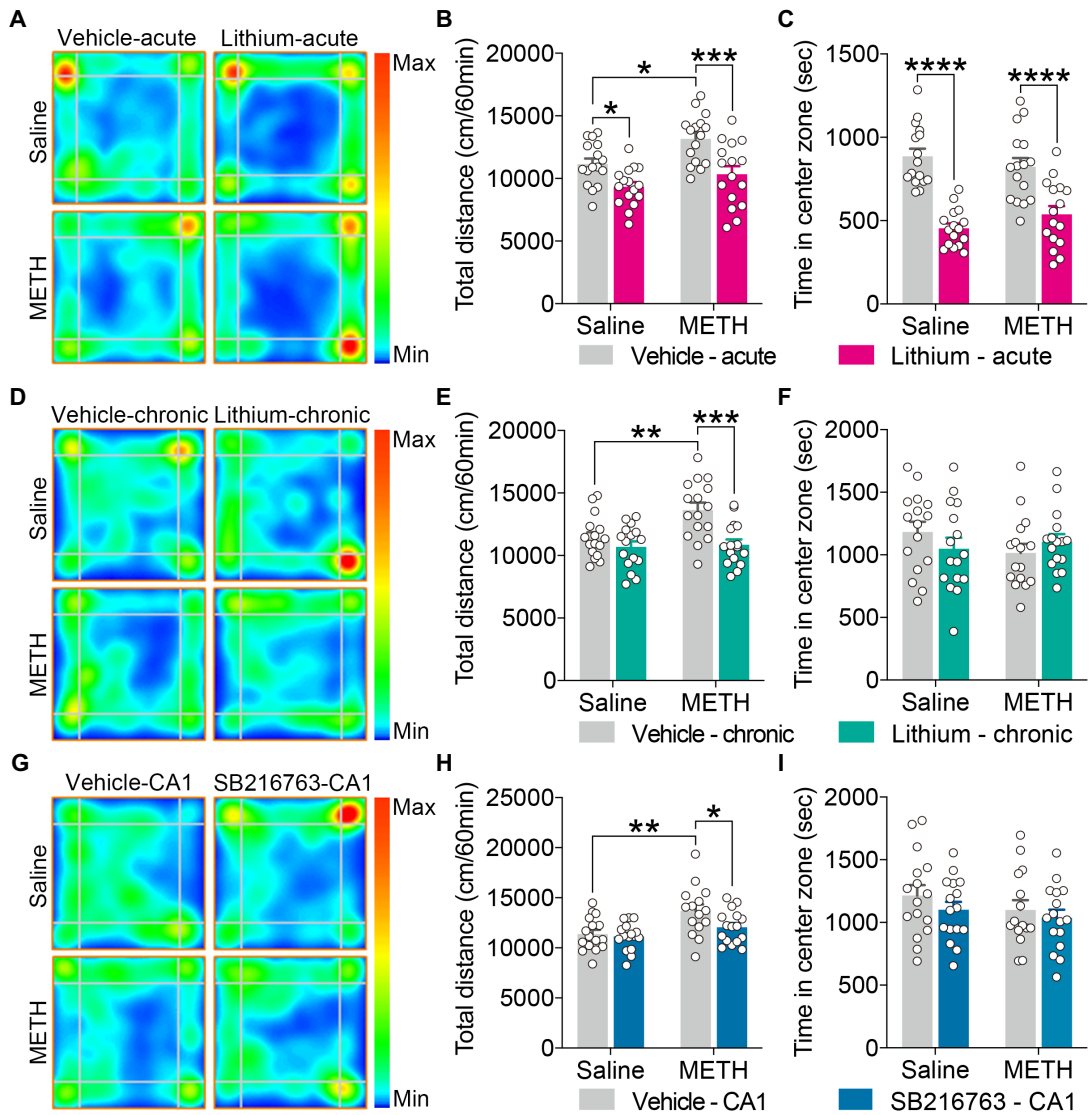
Effect of GSK3β inhibition on adolescent METH exposure-induced hyperactivity in adulthood. Acute systemic intervention **(A–C)**, chronic systemic intervention **(D–F)**, and chronic intervention within CA1 **(G–I)** reduced adolescent METH exposure-induced hyperactivity in adulthood, but Li–acute administration attenuated locomotor activity and led to anxiety-like behavior. Representative heat maps show the location of the mice during the OFT **(A,D,G)**. Histograms show the total distance moved **(B,E,H)** and time spent in the center **(C,F,I)** during the OFT. Data are presented as the mean +/− SEM, each symbol represents the independent of a single animal; two-way ANOVA followed by the Bonferroni *post hoc* test; *n* = 16 ~ 17/group; *p < 0.05, **p < 0.01, ***p < 0.001, and ****p < 0.0001, comparison between the two indicated groups. See also Supplementary Figure S1.

#### Three-chamber social behavior test (sociability and social recognition memory)

The test apparatus was a three-chambered box with two lateral chambers (lateral, 35 × 20 × 30 cm; middle, 35 × 15 × 30 cm). Mice were first allowed to habituate to the apparatus for 5 min, followed by two successive phases (T1 and T2) to investigate their sociability and social recognition memory. In the T1 phase (sociability test), an unfamiliar C57BL/6J male mouse (stranger), which had no prior contact with the subject mouse, was placed into one of the two lateral chambers and enclosed in a circular acrylic cage that only allowed nose contact between the cage bars. Another empty cage of the same design was placed in the other lateral chamber. The subject mice were placed in the central chamber and allowed to freely explore the test apparatus for 10 min. The location of the stranger mouse in the left or right chamber was interchanged between the trials. In the T2 phase (social recognition memory test, a second 10 min period), 30 min after the T1 phase, a second unfamiliar mouse (novel) was placed in the lateral chamber that had been empty in the T1 phase and enclosed in the circular acrylic cage. The subject mouse had a choice between the first, already investigated unfamiliar mouse (stranger (T1), familiar (T2)), and the novel unfamiliar mouse. The interaction was defined as the sniffing or direct contact when the subject animal oriented its nose or initiated physical contact within 2 cm of the stranger/novel mouse contained in the wired cage (Leung et al., 2018). Sociability was expressed using sociability scores that were defined as the difference between the interaction time with the stranger and empty chambers. Time in contact with the stranger (%) was defined as the interaction time with the stranger divided by the total interaction time; entries into the stranger chamber (%) were also recorded. Social recognition memory was expressed using social recognition scores that were defined as the difference between the interaction time with the novel and familiar mice. Time in contact with the novel (%) was defined as the interaction time with the novel animal divided by the total interaction time; entries in the novel chamber (%) were also recorded.

#### Novel object recognition test (object recognition memory)

The test apparatus was a rectangular box (35 × 20 × 30 cm). This test consisted of three phases: habituation, training, and testing (Leger et al., 2013). On day 1 (habituation phase, PND 102), the mice were habituated to the apparatus twice for 10 min each. On day 2 (PND 103), in the training phase, two identical objects were symmetrically fixed to the floor of the apparatus, 10 cm from the walls, and the mice were allowed to freely explore the apparatus for 10 min. In the testing phase, 30 min after the training phase, one of the objects used during the training phase was replaced with a novel object, and the mice were placed back in the apparatus for free exploration for 5 min. The objects used in this test were similar in size but different in color, shape, and texture and had a similar preference for mice. Object exploration was defined as sniffing or touching an object while looking at it at a distance of <2 cm. The object preference in training (%) was defined as the exploration time of one of the two identical objects divided by the total exploration time of all objects, and the novel preference in testing (%) was defined as the exploration time of the novel object divided by the total exploration time of both objects. The total exploration time in the training and testing phases was also recorded.

#### Standard two-trial Y-maze test (spatial recognition memory)

The standard two-trial Y-maze test was used to assess spatial recognition memory (Dellu et al., 2000). This test was also conducted using a Y-maze, which had three identical arms, and one of the three arms was defined as the novel arm. The protocol and data recorded in this test were the same as those of the modified two-trial Y-maze test.

**Figure 3:**
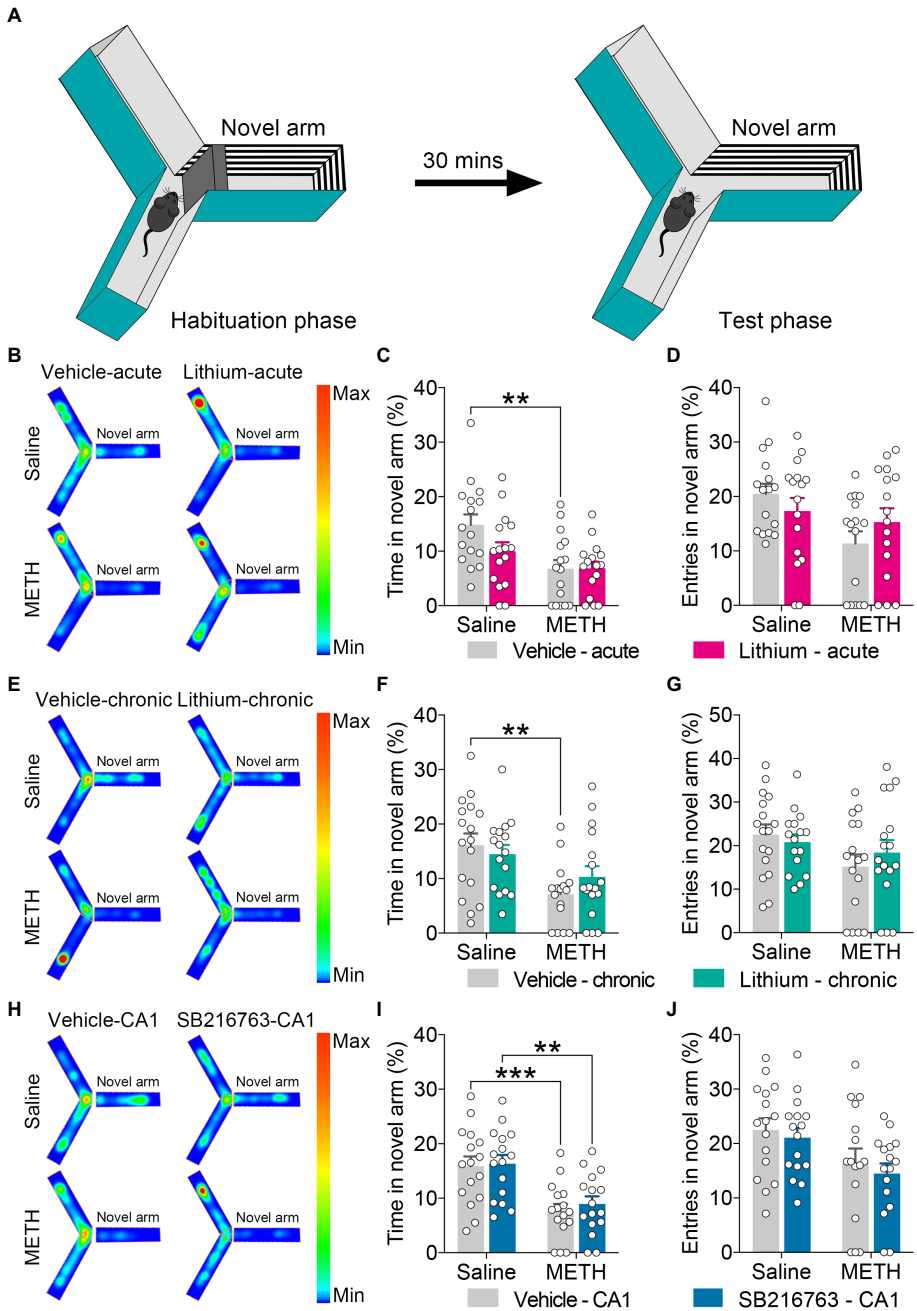
Effect of GSK3β inhibition on adolescent METH exposure-induced novel spatial exploration impairment in adulthood. Apparatus and placements of the mice for the modified two-trial Y-maze test **(A)**. There was no significant influence by the acute systemic intervention **(B–D)**, chronic systemic intervention **(E–G)**, or chronic intervention within CA1 **(H–J)** in adolescent METH exposure-induced novel spatial exploration impairment in adulthood. Representative heat maps show the location of the mice during the testing phase **(B,E,H)**. Histograms show the time spent (%) **(C,F,I)** and entries (%) **(D,G,J)** in the novel arm in this test. Data are presented as the mean +/− SEM, each symbol represents the independent of a single animal; two-way ANOVA followed by the Bonferroni *post hoc* test; *n* = 16 ~ 17/group; **p < 0.01 and ***p < 0.001, comparison between the two indicated groups. See also Supplementary Figure S2.

### Molecular and histological analysis

#### Western blot

Mouse brains were rapidly mounted onto a cryostat, and coronal sections of the DHP CA1 subregion were obtained according to Paxino and Franklin’s Stereotaxic Atlas, 2nd edition (Franklin and Paxinos, 2001); they were then stored at −80°C until processing. Western blotting was conducted as previously described (Wang et al., 2017). The dilutions of primary antibodies were as follows: phosphorylated pGSK3β-S9 (1:1000, Cell Signaling Technology, Beverly, MA, United States), total-GSK3β (t-GSK3β) (1:2000, Cell Signaling Technology), GAPDH (internal control, 1:2000, Pioneer Biotechnology, Xi’an, China). All species-appropriate horseradish peroxidase-conjugated secondary antibodies (Pioneer Biotechnology) were used at a dilution of 1:10,000.

#### Immunohistochemistry

Mice were anesthetized with sodium pentobarbital and intracardially perfused with 4% paraformaldehyde in 0.1 M phosphate buffer (PB; pH 7.4). Brains were immediately removed and post-fixed in 4% paraformaldehyde. After being saturated in 30% (w/v) sucrose in 0.1 M PB buffer (pH 7.4), the brains were serially cut into 20-μm thick transverse sections with a freezing microtome (CM1950, Leica). Immunohistochemical staining was performed according to the manufacturer’s protocol using a Biotin-Streptavidin HRP Detection System (SP-9001, ZSGB-BIO, Beijing, China) (Yan et al., 2019). The dilution of the primary antibody pGSK3β-S9 (Cell Signaling Technology) was 1:100. Images of the processed sections were captured using a Leica MZFL III microscope. The integrated optical density (IOD) in the CA1 subregion of the DHP was evaluated using Image-Pro Plus 6.0 software (IPP, Media Cybernetics, Wokingham, United Kingdom).

#### Transmission electron microscopic analysis

Mice were anesthetized with sodium pentobarbital and intracardially perfused with saline and then with 0.1 M PB buffer (pH 7.4) containing 4% paraformaldehyde and 0.25% glutaraldehyde. Brains were removed immediately and stored in 0.1 M PB buffer (pH 7.4) with 4% paraformaldehyde and 2.5% glutaraldehyde at 4°C. The CA1 subregion of the DHP was extracted and dissected into ~1 mm^3^ pieces. The samples were then fixed in a fresh solution of 1% osmium tetroxide for 90 min, dehydrated in ethanol, and embedded in epon-araldite resin. Ultrathin sections were cut and placed onto grids, stained with 2% aqueous uranyl acetate, and counterstained with 0.3% lead citrate. The sections were imaged using a Hitachi 7,650 electron microscope operated at 80 kV. Synapses were identified by clear pre-and postsynaptic membranes and the presence of synaptic vesicles in the presynaptic terminals. The number of gray type-1 asymmetric synapses (excitatory synapses), the thickness of the PSD at the thickest part, the length of the active zone, and the width of the synaptic cleft in asymmetrical synapses were measured. Quantification was performed using 10 random sections (more than 60 asymmetric synapses) per mouse.

### Statistical analyses

Statistical analyses were performed using GraphPad Prism 9.0 (GraphPad Software Inc., La Jolla, CA, United States) and SPSS 25 (IBM, Armonk, NY, United States). The results are presented as the mean ± SEM. The parametric test (two-way ANOVA with Bonferroni’s *post-hoc* test) was applied when normality and homogeneity of variance assumptions were satisfied; otherwise, the nonparametric test (Kruskal-Wallis with Dunn’s *post-hoc* test) was used. For two-way ANOVA, when there was a statistical interaction were between groups comparisons done; if not, the post-hoc test was performed on the main effect variables that were significant. The investigators were blinded to the allocation of the groups and outcome assessments for all the experiments. All statistically significant differences were defined as *p* < 0.05. Detailed statistics are provided in Supplementary Table S1.

## Results

### The effects of GSK3β activity inhibition on the adolescent METH exposure-induced behavioral and cognitive deficits in adulthood

#### Inhibition of GSK3β activity reduced adolescent METH exposure-induced hyperactivity in adulthood

For the OFT, in the acute systemic intervention (Figure 2A), the adolescent METH × vehicle–acute mice were markedly more active than the saline × vehicle–acute and the METH  ×  Li–acute mice, and saline × Li–acute mice were less active than the saline × vehicle–acute mice (Figure 2B) (two-way ANOVA: *F*_Interaction(1,60)_ = 1.038, *p* = 0.3124, *F*_METH(1,60)_ = 9.279, *p* < 0.01, *F*_Li-acute(1,60)_ = 21.99, *p* < 0.0001). In addition, the saline × Li–acute mice traveled less distance in the center than the saline × vehicle–acute and METH × Li–acute mice (Supplementary Figure S1A) (two-way ANOVA: *F*_Interaction(1,60)_ = 2.486, *p* = 0.1201, *F*_METH(1,60)_ = 6.507, *p* < 0.05, *F*_Li-acute(1,60)_ = 20.21, *p* < 0.0001), and the METH × vehicle–acute mice traveled more distance in the corner than the saline × vehicle–acute mice (Supplementary Figure S1B) (two-way ANOVA: *F*_Interaction(1,60)_ = 1.192, *p* = 0.2794, *F*_METH(1,60)_ = 7.982, *p* < 0.01, *F*_Li-acute(1,60)_ = 3.446, *p* < 0.0638). Furthermore, the saline × Li–acute mice stayed shorter in the center and longer in the corner than saline × vehicle–acute mice, and the METH × Li–acute mice stayed shorter in the center and longer in the corner than METH × vehicle–acute mice (Figure 2C and Supplementary Figure S1C) (two-way ANOVA: time spent in center zone, *F*_Interaction(1,60)_ = 2.640, *p* = 0.1095, *F*_METH(1,60)_ = 0.05235, *p* = 0.8198, *F*_Li-acute(1,60)_ = 62.82, *p* < 0.0001; time spent in corner zone, *F*_Interaction(1,60)_ = 0.2303, *p* = 0.6331, *F*_METH(1,60)_ = 0.7871, *p* = 0.3785, *F*_Li-acute(1,60)_ = 16.63, *p* < 0.001).

**Figure 4:**
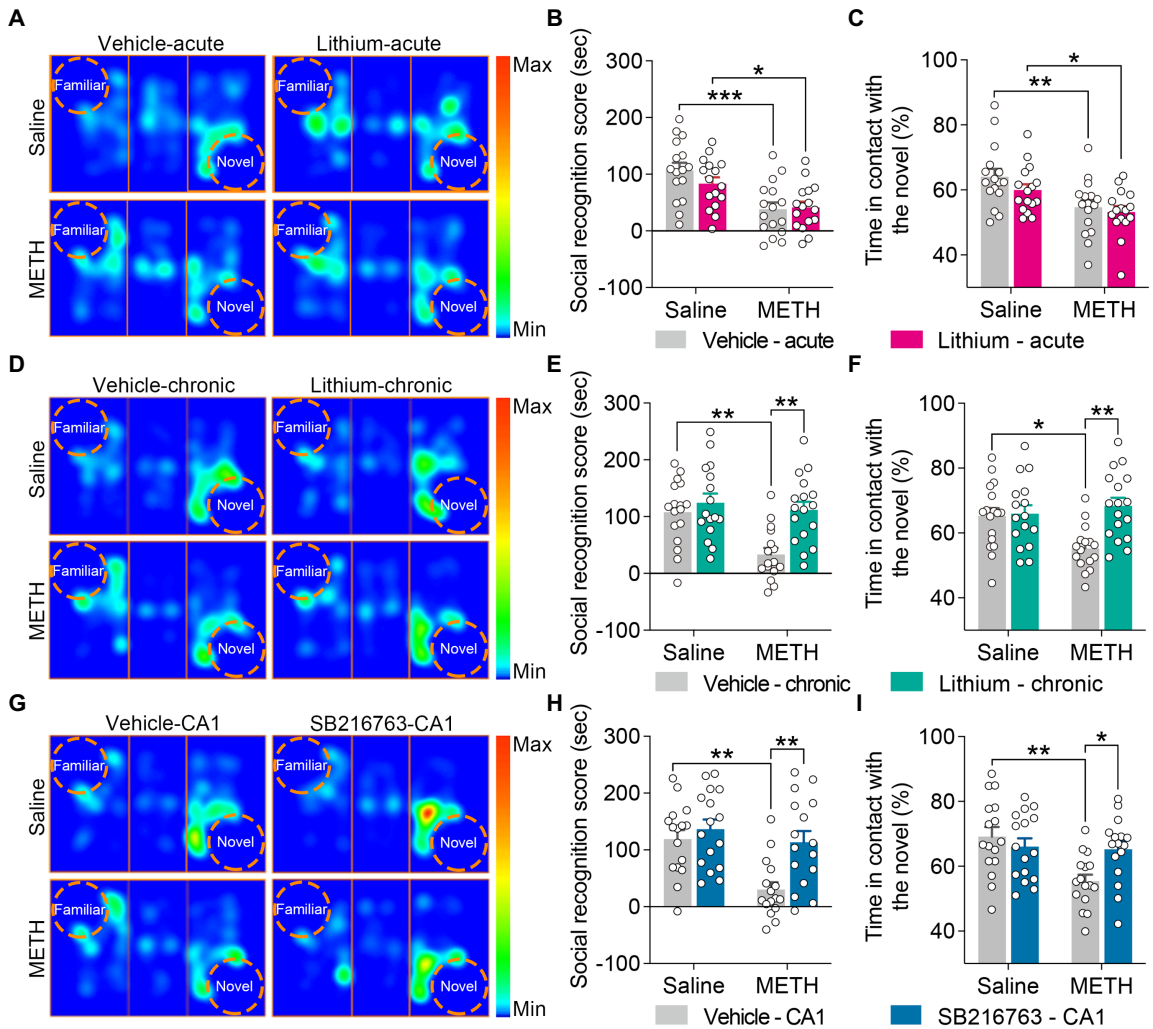
Effect of GSK3β inhibition on adolescent METH exposure-induced social recognition memory impairment in adulthood. Acute systemic intervention **(A–C)** had no significant effects on adolescent METH exposure-induced social behavioral deficit, while chronic systemic intervention **(D–F)** and chronic intervention within CA1 **(G–I)** rescued adolescent METH exposure-induced social recognition memory impairment in adulthood. Representative heat maps show the location of the mice during the social recognition memory test **(A,D,G)**. Histograms show the social recognition score **(B,E,H)** and time in contact with the novel (%) **(C,F,I)** during the social recognition memory test. Data are presented as the mean +/− SEM, each symbol represents the independent of a single animal; two-way ANOVA followed by the Bonferroni *post hoc* test; *n* = 16 ~ 17/group; *p < 0.05, **p < 0.01, and ***p < 0.001, comparison between the two indicated groups. See also Supplementary Figure S3.

In the chronic systemic intervention (Figure 2D), the adolescent METH × vehicle–chronic mice traveled more distance than the saline × vehicle–chronic and METH × Li–chronic mice (Figure 2E) (two-way ANOVA: *F*_Interaction(1,60)_ = 4.781, *p* < 0.05, *F*_METH(1,60)_ = 6.403, *p* < 0.05, *F*_Li-chronic(1,60)_ = 14.13, *p* < 0.001). In addition, the METH × vehicle–chronic mice traveled more distance in the corner than the saline × vehicle–chronic and METH × Li–chronic mice (Supplementary Figure S1E) (two-way ANOVA: *F*_Interaction(1,60)_ = 9.256, *p* < 0.01, *F*_METH(1,60)_ = 7.432, *p* < 0.01, *F*_Li-chronic(1,60)_ = 14.74, *p* < 0.001). There was no statistical significance of the time spent in the center between groups (Figure 2F).

In the chronic CA1 intervention (Figure 2G), the METH × vehicle–CA1 mice traveled a greater distance than the saline × vehicle–CA1 and METH × SB–CA1 mice (Figure 2H) (two-way ANOVA: *F*_Interaction(1,59)_ = 2.223, *p* = 0.1413, *F*_METH(1,59)_ = 12.27, *p* < 0.001, *F*_SB-CA1(1,59)_ = 4.125, *p* < 0.05). Moreover, METH × vehicle–CA1 mice traveled more distance in the corner than saline × vehicle–CA1 and METH × SB–CA1 mice (Supplementary Figure S1H) (Kruskal-Wallis test: *H* = 13.15, *p* < 0.01). There was no statistical significance of the time spent in the center between groups (Figure 2I).

#### No significant effects of inhibition of GSKβ activity on adolescent METH exposure-induced novel spatial exploration impairment

For the modified two-trial Y-maze test, the apparatus and placement of the mice for this test are shown in Figure 3A. In each intervention protocol, adolescent METH-exposed mice spent less time in the novel arm (%) than control mice (Figures 3C,F,I) (two-way ANOVA: acute systemic intervention, *F*_Interaction(1,59)_ = 2.338, *p* = 0.1315, *F*_METH(1,60)_ = 11.89, *p* < 0.01, *F*_Li-acute(1,60)_ = 2.210, *p* = 0.1423; chronic systemic intervention, *F*_Interaction(1,62)_ = 1.693, *p* = 0.1981, *F*_METH(1,62)_ = 12.76, *p* < 0.001, *F*_Li-chronic(1,62)_ = 0.1917, *p* = 0.663; chronic CA1 intervention: *F*_Interaction(1,60)_ = 0.08186, *p* = 0.7758, *F*_METH(1,60)_ = 26.69, *p* < 0.0001, *F*_SB-CA1(1,60)_ = 0.3371, *p* = 0.5637); however, METH × GSKβ inhibitor mice and METH × vehicle mice showed similar characteristics in this test (Figure 3 and Supplementary Figure S2).

#### Chronic treatment with the GSKβ inhibitors ameliorated adolescent METH exposure-induced social recognition memory impairment in adulthood

For the sociability test, two-way ANOVA revealed that all tested mice showed similar sociability characteristics in each intervention protocol (Supplementary Figure S3).

For social recognition memory, in the acute systemic intervention (Figure 4A), the METH × vehicle–acute and METH × Li–acute mice obtained a lower average social recognition score and decreased time in contact with the novel (%) than the saline × vehicle–acute mice (Figures 4B,C) (two-way ANOVA: social recognition score, *F*_Interaction(1,60)_ = 1.326, *p* = 0.254, *F*_METH(1,60)_ = 22.73, *p* < 0.0001, *F*_Li-acute(1,60)_ = 0.7555, *p* = 0.3882; time in contact with the novel, *F*_Interaction(1,60)_ = 0.3973, *p* = 0.5309, *F*_METH(1,60)_ = 15.22, *p* < 0.001, *F*_Li-acute(1,60)_ = 1.839, *p* = 0.1802).

**Figure 5:**
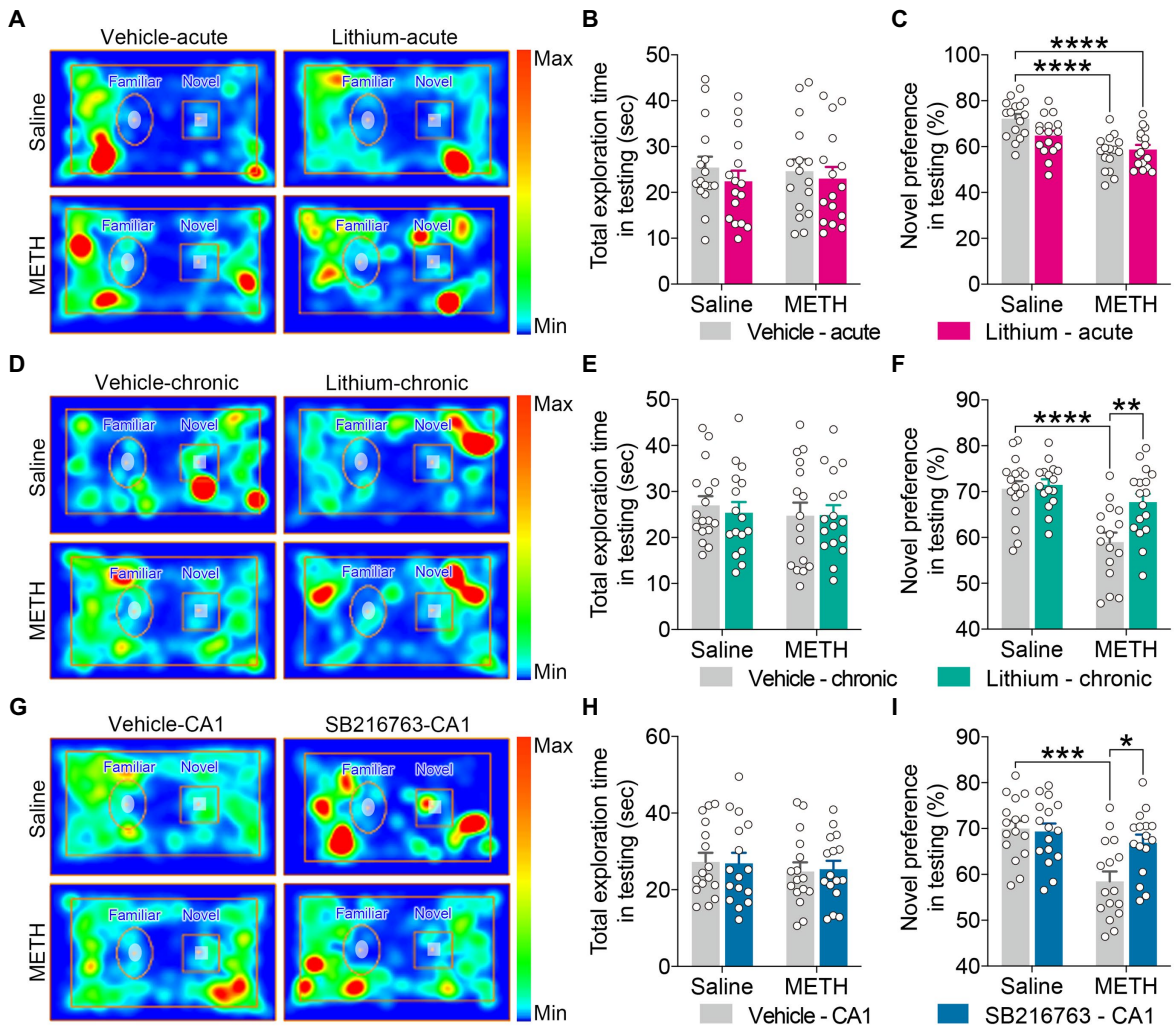
Effect of GSK3β inhibition on adolescent METH exposure-induced object recognition memory impairment in adulthood. Compared with the effect of the acute systemic intervention **(A–C)**, chronic systemic intervention **(D–F)** and chronic intervention within CA1 **(G–I)** improved adolescent METH exposure-induced object recognition memory impairment in adulthood. Representative heat maps show the location of the mice during the testing phase **(A,D,G)**. Histograms show the total exploration time **(B,E,H)** and novel preference (%) **(C,F,I)** during the testing phase. Data are presented as the mean +/− SEM, each symbol represents the independent of a single animal; two-way ANOVA followed by the Bonferroni *post hoc* test; *n* = 16 ~ 17/group; *p < 0.05, **p < 0.01, ***p < 0.001, and ****p < 0.0001, comparison between the two indicated groups. See also Supplementary Figure S4.

In the chronic systemic intervention (Figure 4D), METH × vehicle–chronic mice obtained a lower average social recognition score than saline × vehicle–chronic and METH × Li–chronic mice (Figure 4E) (two-way ANOVA: *F*_Interaction(1,62)_ = 4.850, *p* < 0.05, *F*_METH(1,62)_ = 9.470, *p* < 0.01, *F*_Li-chronic(1,62)_ = 11.34, *p* < 0.01). In addition, METH × vehicle–chronic mice spent less time in contact with the novel (%) than the saline × vehicle–chronic mice (Figure 4F) (two-way ANOVA: *F*_Interaction(1,62)_ = 6.962, *p* < 0.05, *F*_METH(1,62)_ = 2.596, *p* = 0.1122, *F*_Li-chronic(1,62)_ = 8.562, *p* < 0.01).

In the chronic CA1 intervention (Figure 4G), compared to the saline × vehicle–CA1 and METH × SB–CA1 mice, the METH × vehicle–CA1 mice had a lower average social recognition score and spent less time in contact with the novel (%) (Figures 4H,I) (two-way ANOVA: social recognition score, *F*_Interaction(1,60)_ = 4.013, *p* < 0.05, *F*_METH(1,60)_ = 11.59, *p* < 0.01, *F*_SB-CA1(1,60)_ = 9.474, *p* < 0.01; time in contact with the novel, *F*_Interaction(1,60)_ = 6.518, *p* < 0.05, *F*_METH(1,60)_ = 8.344, *p* < 0.01, *F*_SB-CA1(1,60)_ = 1.761, *p* = 0.1896).

#### Chronic treatment with the GSKβ inhibitors improved adolescent METH exposure-induced object recognition memory deficits in adulthood

For the NOR test, in the training phase, all test mice exhibited similar behavioral characteristics in each intervention protocol (Supplementary Figure S4).

In the testing phase, for the total exploration time, no significant differences were observed among the groups for each intervention protocol (Figures 5B,E,H).

**Figure 6:**
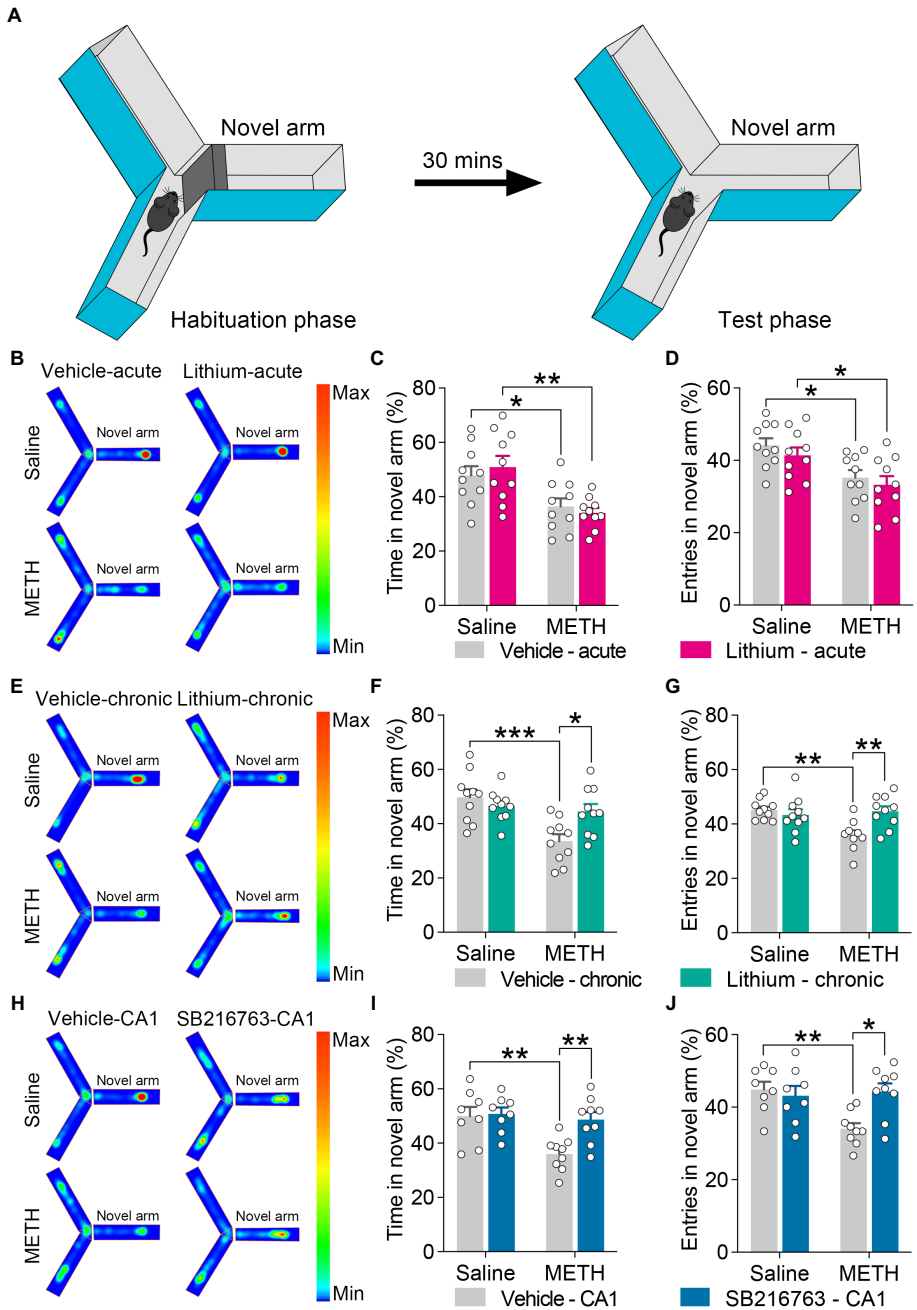
Effect of GSK3β inhibition on adolescent METH exposure-induced spatial recognition memory impairment in adulthood. Apparatus and placements of the mice for the standard two-trial Y-maze test **(A)**. Compared with the effect of the acute systemic intervention **(B–D)**, chronic systemic intervention **(E–G)** and chronic intervention within CA1 **(H–J)** ameliorated adolescent METH exposure-induced spatial recognition memory impairment in adulthood. Representative heat maps show the location of the mice during the testing phase **(B,E,H)**. Histograms show the time spent (%) **(C,F,I)** and entries (%) **(D,G,J)** in the novel arm in this test. Data are presented as the mean +/− SEM, each symbol represents the independent of a single animal; two-way ANOVA followed by the Bonferroni *post hoc* test; *n* = 8 ~ 10/group; *p < 0.05, **p < 0.01, and ***p < 0.001, comparison between the two indicated groups. See also Supplementary Figure S5.

For the exploratory preference of novel objects, in the acute systemic intervention (Figure 5A), the METH × vehicle–acute and METH × Li–acute mice exhibited significantly decreased novel preference (%) compared with the saline × vehicle–acute mice (Figure 5C) (two-way ANOVA: *F*_Interaction(1,60)_ = 4.791, *p* < 0.05, *F*_METH(1,60)_ = 27.00, *p* < 0.0001, *F*_Li-acute(1,60)_ = 2.092, *p* = 0.1533).

In the chronic systemic intervention (Figure 5D), the METH × vehicle–chronic mice showed a significantly decreased novel preference (%) compared to the saline × vehicle–chronic and METH × Li–chronic mice (Figure 5F) (two-way ANOVA: *F*_Interaction(1,62)_ = 5.281, *p* < 0.05, *F*_METH(1,62)_ = 19.99, *p* < 0.0001, *F*_Li-chronic(1,62)_ = 7.543, *p* < 0.01).

In the chronic CA1 intervention (Figure 5G), METH × vehicle–CA1 mice showed significantly decreased novel preference (%) compared to saline × vehicle–CA1 and METH × SB–CA1 mice (Figure 5I) (two-way ANOVA: *F*_Interaction(1,60)_ = 6.040, *p* < 0.05, *F*_METH(1,60)_ = 14.26, *p* < 0.001, *F*_SB-CA1(1,60)_ = 4.433, *p* < 0.05).

#### Chronic treatment with the GSK3β inhibitors ameliorated adolescent METH exposure-induced spatial recognition memory impairment in adulthood

For the standard two-trial Y-maze test, the apparatus and placement of the mice for this test are shown in Figure 6A. In the acute systemic.intervention (Figure 6B), reduced time spent and entries into the novel arm (%) were displayed by METH × vehicle–acute mice and METH × Li–acute mice (Figures 6C,D) (two-way ANOVA: time spent in the novel arm, *F*_Interaction(1,36)_ = 0.7398, *p* = 0.3954, *F*_METH(1,36)_ = 19.83, *p* < 0.0001, *F*_Li-acute(1,36)_ = 0.01305, *p* = 9,097; entries in the novel arm, *F*_Interaction(1,36)_ = 0.02909, *p* = 0.8655, *F*_METH(1,36)_ = 15.52, *p* < 0.001, *F*_Li-acute(1,36)_ = 1.230, *p* = 0.2748).

In the chronic systemic intervention (Figure 6E), the METH × vehicle–chronic mice showed significantly decreased time spent and entries into the novel arm (%) than the saline × vehicle–chronic and METH × Li–chronic mice (Figures 6F,G) (two-way ANOVA: time spent in the novel arm, *F*_Interaction(1,36)_ = 7.652, *p* < 0.01, *F*_METH(1,36)_ = 12.8, *p* < 0.01, *F*_Li-chronic(1,36)_ = 2.303, *p* = 0.1379; entries in the novel arm, *F*_Interaction(1,36)_ = 9.491, *p* < 0.01, *F*_METH(1,36)_ = 5.378, *p* < 0.05, *F*_Li-chronic(1,36)_ = 3.972, *p* = 0.0539).

In the chronic CA1 intervention (Figure 6H), the METH × vehicle–CA1 mice showed significantly decreased time spent and entries into the novel arm (%) compared to saline × vehicle–CA1 and METH × SB–CA1 mice (Figures 6I,J) (two-way ANOVA: time spent in the novel arm, *F*_Interaction(1,30)_ = 4.959, *p* < 0.05, *F*_METH(1,30)_ = 8.989, *p* < 0.01, *F*_SB-CA1(1,30)_ = 6.439, *p* < 0.05; entries in the novel arm, *F*_Interaction(1,30)_ = 7.493, *p* < 0.05, *F*_METH(1,30)_ = 4.911, *p* < 0.05, *F*_SB-CA1(1,30)_ = 3.811, *p* = 0.0603).

Taken together, these results suggest that, in adulthood, all three GSK3β interventions reduce the adolescent METH exposure-induced long-lasting hyperactivity; but only chronic systemic and chronic within CA1 interventions improve that induced social, object, and spatial recognition memory impairments; in addition, acute Li exposure reduces locomotor activity and leads to anxiety-like behavior; for novel spatial exploration impairment, all three interventions have no significant effects.

### Inhibition of GSK3β activity restored adolescent METH exposure-induced increase in the GSK3β activity of the DHP CA1 subregion in adulthood

Previous study reported that in adulthood, increased GSKβ activity of the DHP CA1 subregion may be the reason for behavioral and cognitive impairments induced by adolescent METH exposure, we investigated the effects of GSKβ inhibitors on GSKβ activity in the DHP CA1 subregion (Yan et al., 2019).

**Figure 7:**
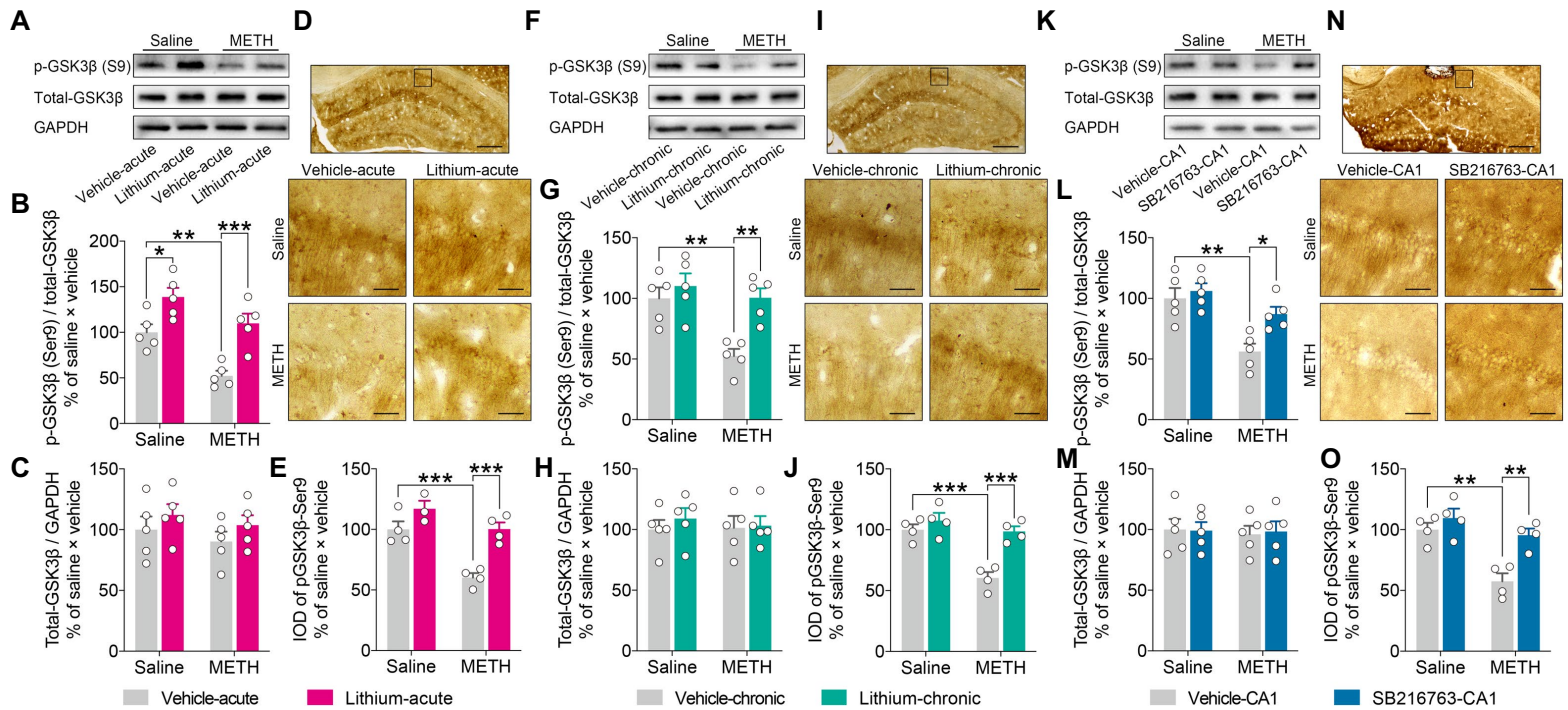
Effects of GSK3β inhibition on abnormal GSK3β activity in the adult DHP CA1 subregion induced by adolescent METH exposure. Acute systemic intervention **(A–E)**, chronic systemic intervention **(F–J)**, and chronic intervention within CA1 **(K–O)** restored the adolescent METH exposure-induced increase in the GSKβ activity of the DHP CA1 subregion in adulthood. Representative western blot of total and phosphorylated GSK3β in the DHP CA1 extract **(A,F,K)**. The relative changes in the ratio of phosphorylation of residues Ser9 (indicative of inactive protein) **(B,G,L)** and the expression of total GSK3β **(C,H,M)** were analyzed. Representative immunostaining of pGSK3β-Ser9 in the DHP CA1. The boxes indicate regions shown at higher magnification in the lower panels; scale bars represent 250 μm under low magnification and 50 μm under high magnification **(D,I,N)**. The relative changes in the IOD of pGSK3β-Ser9 in the DHP CA1 subregion were analyzed **(E,J,O)**. Data are presented as the mean +/− SEM, each symbol represents the independent of a single animal; two-way ANOVA followed by the Bonferroni *post hoc* test; *n* = 5/group in western blot, *n* = 4/group in immunohistochemistry; *p < 0.05, **p < 0.01, and ***p < 0.001, comparison between the two indicated groups.

Western blot analysis (Figures 7A,F,K). demonstrated no changes in the expression level of total-GSK3β in the DHP CA1 subregion among the groups in any intervention protocol (Figures 7C,H,M). However, the adolescent METH-exposed mice showed a significant decrease in the ratio of pGSK3β-Ser9/t-GSK3β compared to the control mice and the METH × GSK3β inhibitor mice in each intervention protocol (Figures 7B,G,L), and Li–acute significantly enhanced the ratio of pGSK3β-Ser9 to t-GSK3β in the DHP CA1 subregion (Figure 7B) (two-way ANOVO: acute systemic intervention: *F*_Interaction(1,16)_ = 1.080, *p* = 0.3141, *F*_METH(1,16)_ = 17.93, *p* < 0.001, *F*_Li-acute(1,16)_ = 28.24, *p* < 0.0001; chronic systemic intervention: *F*_Interaction(1,16)_ = 4.998, *p* < 0.05, *F*_METH(1,16)_ = 11.42, *p* < 0.01, *F*_Li-chronic(1,16)_ = 11.80, *p* < 0.01; chronic CA1 intervention: *F*_Interaction(1,16)_ = 4.763, *p* < 0.05, *F*_METH(1,16)_ = 15.52, *p* < 0.01, *F*_SB-CA1(1,16)_ = 9.219, *p* < 0.01).

Next, immunochemical analysis (Figures 7D,I,N). was performed to confirm the effects of GSK3β inhibitors. In agreement with the results of western blot analysis, adolescent METH-exposed mice showed a significant decrease in the IOD of pGSK3β-Ser9 compared to control mice and METH × GSKβ inhibitor mice in each intervention protocol (Figures 7E,J,O) (two-way ANOVO: acute systemic intervention: *F*_Interaction(1,11)_ = 4.067, *p* = 0.0688, *F*_METH(1,11)_ = 24.47, *p* < 0.001, *F*_Li-acute(1,11)_ = 24.95, *p* < 0.001; chronic systemic intervention: *F*_Interaction(1,12)_ = 9.346, *p* < 0.01, *F*_METH(1,12)_ = 23.30, *p* < 0.001, *F*_Li-chronic(1,12)_ = 20.73, *p* < 0.001; chronic CA1 intervention: *F*_Interaction(1,12)_ = 4.928, *p* < 0.05, *F*_METH(1,12)_ = 19.54, *p* < 0.001, *F*_SB-CA1(1,12)_ = 13.76, *p* < 0.01).

These results suggest that, in adulthood, GSK3β interventions restore adolescent METH exposure-induced long-lasting changes in GSKβ activity in the DHP CA1 subregion.

### Chronic treatment with the GSK3β inhibitors rescued adolescent METH exposure-induced excitatory synaptic ultrastructure alterations of the DHP CA1 subregion in adulthood

**Figure 8:**
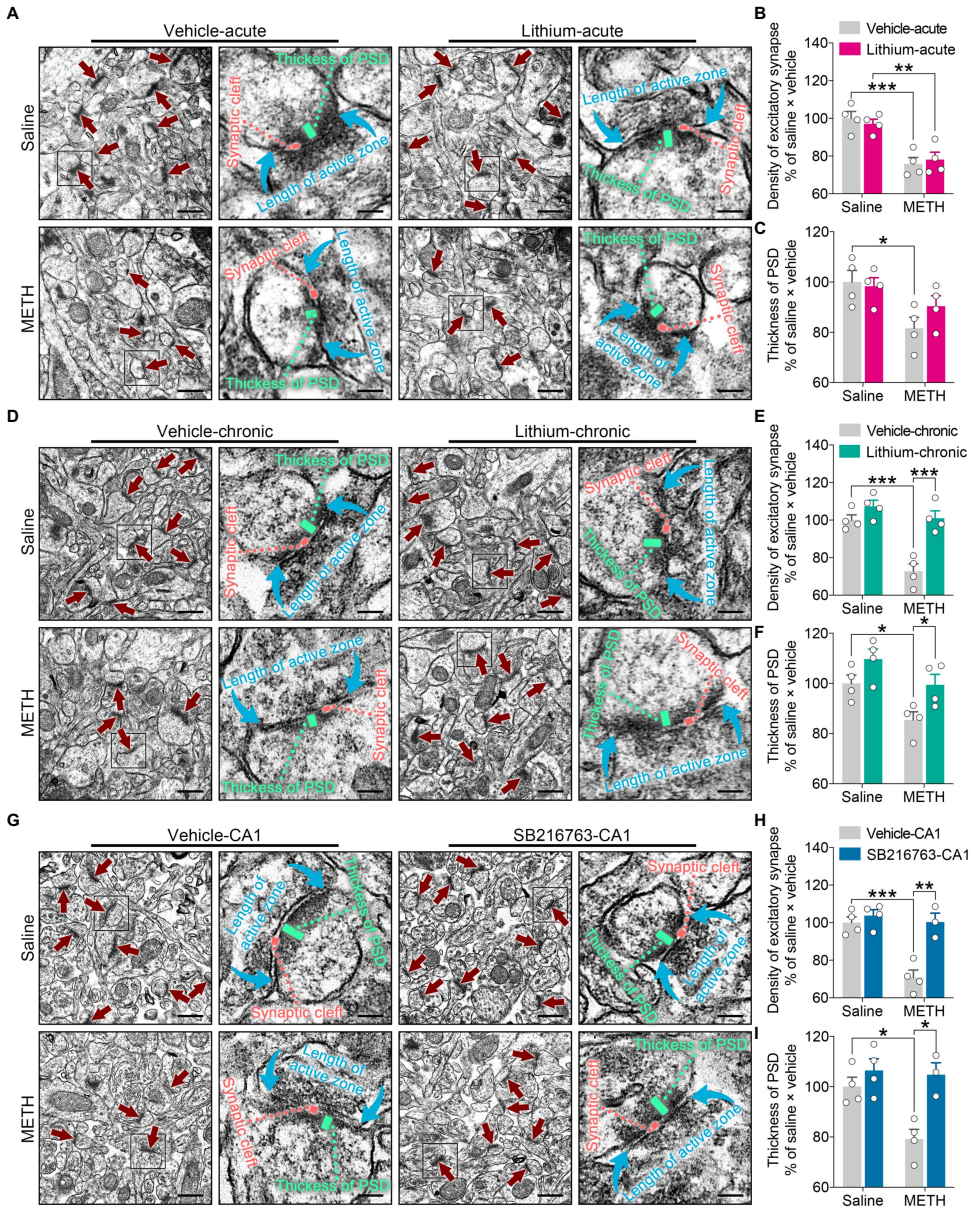
Effects of GSK3β inhibition on synaptic ultrastructural changes in the adult DHP CA1 subregion induced by adolescent METH exposure. Compared with the effect of the acute systemic intervention **(A–C)**, chronic systemic intervention **(D–F)** and chronic intervention within CA1 **(G–I)** resumed adolescent METH exposure-induced alterations in synaptic ultrastructure of the DHP CA1 subregion in adulthood. Representative electron micrographs of the DHP CA1 subregion. The straight crimson arrows indicate Gray’s type-1 asymmetric synapses (excitatory synapses), the boxes indicate regions shown at higher magnification in the lower panels, and scale bars represent 500 nm under low magnification and 100 nm under high magnification **(A,D,G)**. Histograms show the relative changes in the total number of excitatory synapses **(B,E,H)** and the thickness of postsynaptic density (PSD) at the thickest part **(C,F,I)**. More than 60 randomly chosen excitatory synapses from each animal were analyzed. Data are presented as the mean +/− SEM, each symbol represents the independent of a single animal; two-way ANOVA followed by the Bonferroni *post hoc* test; *n* = 4 ~ 5/group; *p < 0.05, **p < 0.01, and ***p < 0.001, comparison between the two indicated groups. See also Supplementary Figure S6.

To determine the structural basis underlying the therapeutic effects of GSK3β inhibition on adolescent METH exposure-induced behavioral and cognitive deficits in adulthood, we examined the synaptic ultrastructure in the DHP CA1 subregion using transmission electron microscopy (Figure 8 and Supplementary Figure S6).

In the acute systemic intervention, reduced excitatory synapse density and PSD thickness in the CA1 subregion were displayed by METH × vehicle–acute mice compared with saline × vehicle–acute mice, and reduced excitatory synapse density in the CA1 subregion was also displayed by METH × Li–acute mice compared with saline × Li–acute mice (Figures 8A–C) (two-way ANOVA: density of excitatory synapses, *F*_Interaction(1,12)_ = 0.5632, *p* = 0.4674, *F*_METH(1,12)_ = 40.01, *p* < 0.0001, *F*_Li-acute(1,12)_ = 0.01149, *p* = 0.9164; PSD thickness, *F*_Interaction(1,12)_ = 1.599, *p* = 0.23, *F*_METH(1,12)_ = 10.02, *p* < 0.01, *F*_Li-acute(1,12)_ = 0.7341, *p* = 0.4083).

In the chronic systemic intervention, the METH × vehicle–chronic mice showed significantly decreased excitatory synapse density and PSD thickness in the CA1 subregion compared to the saline × vehicle–chronic and METH × Li–chronic mice (Figures 8D–F) (two-way ANOVA: density of excitatory synapses, *F*_Interaction(1,12)_ = 9.045, *p* < 0.05, *F*_METH(1,12)_ = 23.16, *p* < 0.001, *F*_Li-chronic(1,12)_ = 26.14, *p* < 0.001; PSD thickness, *F*_Interaction(1,12)_ = 0.3332, *p* = 0.5745, *F*_METH(1,12)_ = 11.23, *p* < 0.01, *F*_Li-chronic(1,12)_ = 10.27, *p* < 0.01).

In the chronic CA1 intervention, the METH × vehicle–CA1 mice showed significantly decreased excitatory synapse density and PSD thickness in the CA1 subregion compared with the saline × vehicle–CA1 and METH × SB–CA1 mice (Figures 8G–I) (two-way ANOVA: density of excitatory synapses, *F*_Interaction(1,11)_ = 12.03, *p* < 0.01, *F*_METH(1,11)_ = 19.30, *p* < 0.01, *F*_SB-CA1(1,11)_ = 20.12, *p* < 0.0001; PSD thickness, *F*_Interaction(1,11)_ = 5.084, *p* < 0.05, *F*_METH(1,11)_ = 6.977, *p* < 0.05, *F*_SB-CA1(1,11)_ = 14.18, *p* < 0.01).

These results suggest that, compared with acute treatment, chronic treatment with the GSK3β inhibitors is more effectiveness in the adolescent METH exposure-induced excitatory synaptic ultrastructural alterations of the DHP CA1 subregion in adulthood.

## Discussion

Developmental METH exposure causes long-lasting neuropsychiatric consequences (Teixeira-Gomes et al., 2015). To the best of our knowledge, this is the first study to investigate how to improve adolescent METH exposure-associated behavioral and cognitive alterations in adulthood. We demonstrated that treatment with a GSK3β inhibitor in adulthood significantly ameliorated adolescent METH exposure-induced long-term deficits in locomotor activity and recognition memory by reversing the aberrant GSK3β activity and synaptic ultrastructure in the DHP CA1 subregion.

Glycogen synthase kinase-3β has extensive biological functions, and abnormal regulation of GSK3β has been observed in the onset and progression of different conditions (Beurel et al., 2015). GSK3β inhibition is not only an effective therapy for several neurological and psychiatric disorders, but is also beneficial for addictive drug-induced neurotoxicity (Takahashi-Yanaga, 2013; Barr and Unterwald, 2020). Therefore, many GSK3β-targeted pharmacological agents are being evaluated in preclinical and clinical trials (Demuro et al., 2021). In this study, we verified two GSK3β inhibitors: LiCl and SB216763, we found both agents were promising for treating adolescent METH exposure-induced behavioral and cognitive deficits in adulthood. It is well known that establishing adequate administration protocol is critical to correctly evaluate the therapeutical effects of drugs (Kim et al., 2008; Wang et al., 2021; Hiratsu et al., 2022). Herein, we performed acute (systemic) and chronic (systemic and within CA1) intervention protocols, and the results revealed that both protocols had a certain effect, but chronic intervention seemed to be better. Specifically, both protocols were effective against adolescent METH exposure-induced hyperactivity in adulthood, these results further highlight the efficacy of GSK3β-targeted therapeutic intervention in hyperactivity-associated behaviors (Mines, 2013). Compared with the ventral hippocampus, the DHP plays a more important role in locomotion (Fanselow and Dong, 2010), and local lesions of serotonin projections into DHP reduced amphetamine-induced locomotor hyperactivity (Kusljic and van den Buuse, 2004). Serotonin regulates the GSK3B activity by type 1 and type 2 serotonin receptors, in turn, GSK3β selectively interacts with 5-hydroxytryptamine-1B receptors (5-HT1BR) for modulating 5-HT1BR activity (Li and Polter, 2011). Thus, we presumed that rebalancing of the serotonin system in the DHP CA1 subregion may be one reason for improving hyperactivity by GSK3B activity inhibition. Nevertheless, for social, object, and spatial recognition memory impairments, only the chronic intervention had a positive impact.

The hippocampus is a complex brain structure that plays a vital role in memory (Borczyk et al., 2021). GSK3β has been regarded as a switch for synaptic plasticity, and hyperactivation of GSK3β is associated with memory deficiencies (Salcedo-Tello et al., 2011). Hypoxic brain damage is one of the most common manifestations of METH exposure, and among the various brain regions, the hippocampus is more vulnerable to hypoxia; thus, adolescent METH exposure may induce long-term hippocampal damage by disturbing development (Zhu et al., 2012; Weaver et al., 2014). Liang et al. (2022) recently reported that adult mice exposed to METH in adolescence had abnormal changes in the structural plasticity of the DHP. Yan et al. (2019) also found adolescent METH exposure caused decreased excitatory synapse density and PSD thickness of the DHP in adulthood, which were predominantly located in the CA1 subregion rather than in the CA3 and DG subregions. Thus, recovering synaptic ultrastructural alterations in the DHP CA1 subregion should be effective against adolescent METH exposure-induced cognitive and behavioral impairments in adulthood. However, our results indicated transient inhibition of GSK3β activity was not sufficient to resume synaptic ultrastructural alterations, which may explain why the chronic intervention is more effective than acute intervention for recognition memory impairment.

Lithium has been used for more than 70 years as one of the most effective agents for the treatment of major mood disorders (Gómez-Sintes and Lucas, 2010). However, because of the narrow therapeutic window index, several side effects of lithium maintenance treatment have been reported in clinical studies, including polyuria, polydipsia, tremor, and weight gain (Gitlin, 2016; Schoot et al., 2020). In this study, we used medium doses of GSK3β inhibitors, and previous studies reported that these dosages significantly inhibited GSK3β activity but rarely caused behavioral and cognitive deficits (Mines, 2013; Xing et al., 2016; Nguyen et al., 2017; Pan et al., 2018; Xiang et al., 2021). Accordingly, our results indicated that chronic GSK3β intervention (systemic and within CA1) did not affect locomotor activity, recognition memory, or CA1 synaptic ultrastructure during the treatment phase. However, acute systemic GSK3β intervention decreased locomotor activity and induced anxiety-like behavior in the OFT. Previously, striatal dopaminergic circuits were implicated in locomotor activity and anxiety disorders (Macpherson and Hikida, 2019; Casado-Sainz et al., 2022), while striatal physiology is affected preferentially by GSK3β inhibition compared with some other brain regions (Gómez-Sintes and Lucas, 2010); thus, the acute side effects may be due to the targeted effects of systemic administration of LiCl on the striatum. Anxiety-like behavior may also be associated with the aversive effect of LiCl, as LiCl was a classical agent for inducing condition place aversion (Cloutier et al., 2018). Therefore, the potential side effects of GSK3β inhibitors should be carefully considered when treating adult deficits associated with adolescent METH abuse, and brain region-targeting therapy may be a good strategy.

We used the modified two-trial Y-maze test to detect novel spatial exploration behavior; however, our results showed that both acute and chronic GSK3β interventions had no significant therapeutic effects on adolescent METH exposure-induced novel spatial exploration impairment in adulthood. Novel spatial exploration critically depends on the intact function of novel exploration and recognition memory (Melnikova et al., 2006; Szalardy et al., 2018). To further confirm which function was more difficult to improve by GSK3β inhibition, we performed a standard two-trial Y-maze test in a separate cohort. We verified that chronic GSK3β intervention rescued adolescent METH exposure-induced spatial recognition memory impairment in adulthood. Moreover, the mice avoided the novel arm in the modified two-trial Y-maze test because the average time spent in the novel arm (%) was less than one-third, which is typical if the mice have no preference for each arm (Dellu et al., 2000). Anxiety also gives rise to exploration behavioral deficits; however, previous study confirmed that adult mice with adolescent METH exposure did not exhibit anxiety-like behavior in a similar time window (Yan et al., 2019). In the modified two-trial Y-maze test, to increase recognition and navigation in the novel space, the wall of the novel arm was marked integrally with a black-and-white stripe that was entirely different from the other arms, and we speculated that this novel space would generate mild stress in mice, causing avoidance behavior (Aylward et al., 2019; Vogel and Schwabe, 2019; Kondev et al., 2022). The stress-related avoidance behavior in this study may be because some other brain regions are also damaged by adolescent METH exposure, which is difficult to ameliorate by GSK3β inhibition in adulthood. Further studies are required to confirm these results.

This study has three limitations that must be addressed. First, LiCl and SB216763 directly inhibit GSK3α and GSK3β. Although GSK3α is involved in fewer signaling pathways than GSK3β, and its expression level is relatively lower and decreases with age (KEGG pathway)^1^ (Giese, 2009), the effect of GSK3α cannot be eliminated in the present study. Second, although LiCl and SB216763 could rescue hyperactivation of GSK3β in several disease-associated rodent models (Maixner and Weng, 2013; Barr and Unterwald, 2020), in the present study, there was still an interval of several days between behavioral tests and the molecular and ultrastructural analysis, suggesting that alterations in molecular and synaptic plasticity may not perfectly reflect behavioral alterations. Third, this study is still a preclinical work, mice cannot fully mimic human drug abuse behavior, and the side effects of GSK3β inhibitor remain elusive, investigating GSK3β inhibitor-related toxicity, behavioral and cognitive alterations is necessary in future studies.

In summary, the present results revealed that chronic GSK3β inhibition attenuates chronic METH exposure-induced hyperactivity and recognition memory impairment by rescuing synaptic ultrastructural abnormalities in the DHP CA1 subregion in adulthood. This extends the scope of potential applications of GSK3β inhibitors and suggests that chronic administration of GSK3β inhibitors maybe an option for treating behavioral and cognitive deficits associated with adolescent METH abuse in adulthood.

## Data availability statement

The original contributions presented in the study are included in the article/Supplementary material, further inquiries can be directed to the corresponding authors.

## Ethics statement

The animal study was reviewed and approved by the Institutional Animal Care and Use Committee of Xi’an Jiaotong University.

## Author contributions

PY, SW, and JLa conceived and designed the study. PY, JLi, HM, YF, JC, and YB performed the experiments and acquired the data. XH, YZ, and SW provided technical support and analyzed the data. PY and JLa wrote the manuscript. All authors contributed to the article and approved the submitted version.

## Funding

This work was supported by the National Natural Science Foundation of China (Grant Nos. 82001999 and 82171880), the China Postdoctoral Science Foundation (Grant No. 2020M673422), and the NHC Key Laboratory of Forensic Science (Xi’an Jiaotong University) Open Projects Fund (Grant No. 2020FYXH002).

## Conflict of interest

The authors declare that the research was conducted in the absence of any commercial or financial relationships that could be construed as a potential conflict of interest.

## Publisher’s note

All claims expressed in this article are solely those of the authors and do not necessarily represent those of their affiliated organizations, or those of the publisher, the editors and the reviewers. Any product that may be evaluated in this article, or claim that may be made by its manufacturer, is not guaranteed or endorsed by the publisher.

## Footnotes

^1^ http://www.genome.jp/kegg/pathway.html

## References

[ref1] Aylward J. Valton V. Ahn W.-Y. Bond R. L. Dayan P. Roiser J. P.. (2019). Altered learning under uncertainty in unmedicated mood and anxiety disorders. Nat. Hum. Behav. 3, 1116–1123. doi: 10.1038/s41562-019-0628-0, PMID: 31209369 PMC6790140

[ref2] Barr J. L. Unterwald E. M. (2020). Glycogen synthase kinase-3 signaling in cellular and behavioral responses to psychostimulant drugs. Biochim. Biophys. Acta, Mol. Cell Res. 1867:118746. doi: 10.1016/j.bbamcr.2020.118746, PMID: 32454064 PMC7313643

[ref3] Basedow L. A. Kuitunen-Paul S. Wiedmann M. F. Ehrlich S. Roessner V. Golub Y. (2021). Verbal learning impairment in adolescents with methamphetamine use disorder: a cross-sectional study. BMC Psychiatry 21:166. doi: 10.1186/s12888-021-03169-3, PMID: 33765981 PMC7993453

[ref4] Beurel E. Grieco S. F. Jope R. S. (2015). Glycogen synthase kinase-3 (GSK3): regulation, actions, and diseases. Pharmacol. Ther. 148, 114–131. doi: 10.1016/j.pharmthera.2014.11.016, PMID: 25435019 PMC4340754

[ref5] Bhat R. V. Andersson U. Andersson S. Knerr L. Bauer U. Sundgren-Andersson A. K. (2018). The conundrum of GSK3 inhibitors: is it the dawn of a new beginning? J. Alzheimers Dis. 64, S547–S554. doi: 10.3233/JAD-179934, PMID: 29758944

[ref6] Borczyk M. Radwanska K. Giese K. P. (2021). The importance of ultrastructural analysis of memory. Brain Res. Bull. 173, 28–36. doi: 10.1016/j.brainresbull.2021.04.019, PMID: 33984429

[ref7] Brust V. Schindler P. M. Lewejohann L. (2015). Lifetime development of behavioural phenotype in the house mouse (*Mus musculus*). Front. Zool. 12:S17. doi: 10.1186/1742-9994-12-s1-s17 26816516 PMC4722345

[ref8] Casado-Sainz A. Gudmundsen F. Baerentzen S. L. Lange D. Ringsted A. Martinez-Tejada I.. (2022). Dorsal striatal dopamine induces fronto-cortical hypoactivity and attenuates anxiety and compulsive behaviors in rats. Neuropsychopharmacology 47, 454–464. doi: 10.1038/s41386-021-01207-y, PMID: 34725486 PMC8559920

[ref9] Chen X. Sun G. Tian E. Zhang M. Davtyan H. Beach T. G.. (2021). Modeling sporadic Alzheimer's disease in human brain organoids under serum exposure. Adv. Sc. 8, –e2101462. doi: 10.1002/advs.202101462, PMID: 34337898 PMC8456220

[ref10] Cloutier C. J. Kavaliers M. Ossenkopp K.-P. (2018). Lipopolysaccharide (LPS) induced sickness in adolescent female rats alters the acute-phase response and lithium chloride (LiCl)- induced impairment of conditioned place avoidance/aversion learning, following a homotypic LPS challenge in adulthood. Behav. Brain Res. 351, 121–130. doi: 10.1016/j.bbr.2018.05.033, PMID: 29885379

[ref11] Dellu F. Contarino A. Simon H. Koob G. F. Gold L. H. (2000). Genetic differences in response to novelty and spatial memory using a two-trial recognition task in mice. Neurobiol. Learn. Mem. 73, 31–48. doi: 10.1006/nlme.1999.3919, PMID: 10686122

[ref12] Demuro S. Di Martino R. M. C. Ortega J. A. Cavalli A. (2021). GSK-3β, FYN, and DYRK1A: master regulators in neurodegenerative pathways. Int. J. Mol. Sci. 22:9098. doi: 10.3390/ijms22169098, PMID: 34445804 PMC8396491

[ref13] Fanselow M. S. Dong H. W. (2010). Are the dorsal and ventral hippocampus functionally distinct structures? Neuron 65, 7–19. doi: 10.1016/j.neuron.2009.11.031, PMID: 20152109 PMC2822727

[ref14] Franklin K.B.J. Paxinos G. (2001). The mouse brain in stereotaxic coordinates. San Diego, CA: Academic Press.

[ref15] Fuhrmann D. Knoll L. J. Blakemore S.-J. (2015). Adolescence as a sensitive period of brain development. Trends Cogn. Sci. 19, 558–566. doi: 10.1016/j.tics.2015.07.008 26419496

[ref16] García-Cabrerizo R. Bis-Humbert C. García-Fuster M. J. (2018). Methamphetamine binge administration during late adolescence induced enduring hippocampal cell damage following prolonged withdrawal in rats. Neurotoxicology 66, 1–9. doi: 10.1016/j.neuro.2018.02.016, PMID: 29501631

[ref100] Giese K. P. (2009). GSK-3: a key player in neurodegeneration and memory. IUBMB Life, 61:516–521. doi: 10.1002/iub.187, PMID: 19391164

[ref17] Gitlin M. (2016). Lithium side effects and toxicity: prevalence and management strategies. Int. J. Bipolar Disord. 4:27. doi: 10.1186/s40345-016-0068-y, PMID: 27900734 PMC5164879

[ref18] Gómez-Sintes R. Lucas J. J. (2010). NFAT/Fas signaling mediates the neuronal apoptosis and motor side effects of GSK-3 inhibition in a mouse model of lithium therapy. J. Clin. Invest. 120, 2432–2445. doi: 10.1172/JCI37873, PMID: 20530871 PMC2898581

[ref19] Gyu H. J. Hyun K. D. Hwan L. C. Se Jin P. Jong Min K. Mudan C.. (2012). GSK-3β activity in the hippocampus is required for memory retrieval. Neurobiol. Learn. Mem. 98, 122–129. doi: 10.1016/j.nlm.2012.07.003 22800848

[ref20] Hiratsu A. Tataka Y. Namura S. Nagayama C. Hamada Y. Miyashita M. (2022). The effects of acute and chronic oral L-arginine supplementation on exercise-induced ammonia accumulation and exercise performance in healthy young men: a randomised, double-blind, cross-over, placebo-controlled trial. J. Exerc. Sci. Fitness 20, 140–147. doi: 10.1016/j.jesf.2022.02.003, PMID: 35308069 PMC8904605

[ref21] Jung S. Kim Y. Kim M. Seo M. Kim S. Kim S.. (2021). Exercise pills for drug addiction: forced moderate endurance exercise inhibits methamphetamine-induced hyperactivity through the striatal glutamatergic signaling pathway in male Sprague Dawley rats. Int. J. Mol. Sci. 22:8203. doi: 10.3390/ijms22158203, PMID: 34360969 PMC8348279

[ref22] Kamei H. Nagai T. Nakano H. Togan Y. Takayanagi M. Takahashi K.. (2006). Repeated methamphetamine treatment impairs recognition memory through a failure of novelty-induced ERK1/2 activation in the prefrontal cortex of mice. Biol. Psychiatry 59, 75–84. doi: 10.1016/j.biopsych.2005.06.006, PMID: 16139811

[ref23] Kim K.-H. Oudit G. Y. Backx P. H. (2008). Erythropoietin protects against doxorubicin-induced cardiomyopathy via a phosphatidylinositol 3-kinase-dependent pathway. J. Pharmacol. Exp. Ther. 324, 160–169. doi: 10.1124/jpet.107.125773, PMID: 17928571

[ref24] King M. K. Pardo M. Cheng Y. Downey K. Jope R. S. Beurel E. (2014). Glycogen synthase kinase-3 inhibitors: rescuers of cognitive impairments. Pharmacol. Ther. 141, 1–12. doi: 10.1016/j.pharmthera.2013.07.010, PMID: 23916593 PMC3867580

[ref25] Kondev V. Morgan A. Najeed M. Winters N. D. Kingsley P. J. Marnett L.. (2022). The endocannabinoid 2-Arachidonoylglycerol Bidirectionally modulates acute and protracted effects of predator odor exposure. Biol. Psychiatry 92, 739–749. doi: 10.1016/j.biopsych.2022.05.012, PMID: 35961791 PMC9827751

[ref26] Kusljic S. van den Buuse M. (2004). Functional dissociation between serotonergic pathways in dorsal and ventral hippocampus in psychotomimetic drug-induced locomotor hyperactivity and prepulse inhibition in rats. Eur. J. Neurosci. 20, 3424–3432. doi: 10.1111/j.1460-9568.2004.03804.x, PMID: 15610175

[ref27] Lee J. H. Kim D. G. (2019). Diminished food-related motivation in adult rats treated with methamphetamine during adolescence. Neuroreport 30, 1143–1147. doi: 10.1097/WNR.0000000000001325, PMID: 31568209 PMC6855325

[ref28] Leger M. Quiedeville A. Bouet V. Haelewyn B. Boulouard M. Schumann-Bard P.. (2013). Object recognition test in mice. Nat. Protoc. 8, 2531–2537. doi: 10.1038/nprot.2013.155 24263092

[ref29] Leung C. Kim J. C. Jia Z. (2018). Three-chamber social approach task with Optogenetic stimulation (mice). Biol. Protoc. 8:e3120:e3120. doi: 10.21769/BioProtoc.3120, PMID: 34532561 PMC8342085

[ref30] Li X. Polter A. (2011). Glycogen synthase Kinase-3 is an intermediate modulator of serotonin neurotransmission. Front. Mol. Neurosci. 4:31. doi: 10.3389/fnmol.2011.00031, PMID: 22028682 PMC3199786

[ref31] Liang M. Zhu L. Wang R. Su H. Ma D. Wang H.. (2022). Methamphetamine exposure in adolescent impairs memory of mice in adulthood accompanied by changes in neuroplasticity in the dorsal hippocampus. Front. Cell. Neurosci. 16:892757. doi: 10.3389/fncel.2022.892757, PMID: 35656409 PMC9152172

[ref32] Lin L. Cao J. Yang S.-S. Fu Z.-Q. Zeng P. Chu J.. (2018). Endoplasmic reticulum stress induces spatial memory deficits by activating GSK-3. J. Cell. Mol. Med. 22, 3489–3502. doi: 10.1111/jcmm.13626, PMID: 29675957 PMC6010738

[ref33] Luikinga S. J. Kim J. H. Perry C. J. (2018). Developmental perspectives on methamphetamine abuse: exploring adolescent vulnerabilities on brain and behavior. Prog. Neuro Psychopharmacol. Biol. Psychiatry 87, 78–84. doi: 10.1016/j.pnpbp.2017.11.010, PMID: 29128447

[ref34] Macpherson T. Hikida T. (2019). Role of basal ganglia neurocircuitry in the pathology of psychiatric disorders. Psychiatry Clin. Neurosci. 73, 289–301. doi: 10.1111/pcn.12830, PMID: 30734985

[ref35] Maixner D. W. Weng H. R. (2013). The role of glycogen synthase kinase 3 Beta in neuroinflammation and pain. J. Pharm. Pharmacol. 1:001. doi: 10.13188/2327-204X.1000001, PMID: 25309941 PMC4193379

[ref36] Melnikova T. Savonenko A. Wang Q. Liang X. Hand T. Wu L.. (2006). Cycloxygenase-2 activity promotes cognitive deficits but not increased amyloid burden in a model of Alzheimer's disease in a sex-dimorphic pattern. Neuroscience 141, 1149–1162. doi: 10.1016/j.neuroscience.2006.05.001, PMID: 16753269

[ref37] Mines M. A. (2013). Hyperactivity: glycogen synthase kinase-3 as a therapeutic target. Eur. J. Pharmacol. 708, 56–59. doi: 10.1016/j.ejphar.2013.02.055, PMID: 23500205

[ref38] Nazari A. Perez-Fernandez C. Flores P. Moreno M. Sánchez-Santed F. (2020). Age-dependent effects of repeated methamphetamine exposure on locomotor activity and attentional function in rats. Pharmacol. Biochem. Behav. 191:172879. doi: 10.1016/j.pbb.2020.172879, PMID: 32088359

[ref39] Neuwirth L. S. Verrengia M. T. Harikinish-Murrary Z. I. Orens J. E. Lopez O. E. (2022). Under or absent reporting of light stimuli in testing of anxiety-like behaviors in rodents: the need for standardization. Front. Mol. Neurosci. 15:912146. doi: 10.3389/fnmol.2022.912146, PMID: 36061362 PMC9428565

[ref40] Nguyen T. Fan T. George S. R. Perreault M. L. (2017). Disparate effects of lithium and a GSK-3 inhibitor on neuronal oscillatory activity in prefrontal cortex and hippocampus. Front. Aging Neurosci. 9:434. doi: 10.3389/fnagi.2017.00434, PMID: 29375364 PMC5770585

[ref41] North A. Swant J. Salvatore M. F. Gamble-George J. Prins P. Butler B.. (2013). Chronic methamphetamine exposure produces a delayed, long-lasting memory deficit. Synapse 67, 245–257. doi: 10.1002/syn.21635, PMID: 23280858 PMC3831527

[ref42] Ochoa E. L. M. (2022). Lithium as a neuroprotective agent for bipolar disorder: an overview. Cell. Mol. Neurobiol. 42, 85–97. doi: 10.1007/s10571-021-01129-9, PMID: 34357564 PMC11441275

[ref43] Pan Y. Short J. L. Newman S. A. Choy K. H. C. Tiwari D. Yap C.. (2018). Cognitive benefits of lithium chloride in APP/PS1 mice are associated with enhanced brain clearance of β-amyloid. Brain Behav. Immun. 70, 36–47. doi: 10.1016/j.bbi.2018.03.007, PMID: 29545118

[ref44] Salcedo-Tello P. Ortiz-Matamoros A. Arias C. (2011). GSK3 function in the brain during development, neuronal plasticity, and neurodegeneration. Int. J. Alzheimers Dis. 2011:189728. doi: 10.4061/2011/189728, PMID: 21660241 PMC3109514

[ref45] Schoot T. S. Molmans T. H. J. Grootens K. P. Kerckhoffs A. P. M. (2020). Systematic review and practical guideline for the prevention and management of the renal side effects of lithium therapy. Eur. Neuropsychopharmacol. 31, 16–32. doi: 10.1016/j.euroneuro.2019.11.006, PMID: 31837914

[ref46] Shukla M. Vincent B. (2021). Methamphetamine abuse disturbs the dopaminergic system to impair hippocampal-based learning and memory: an overview of animal and human investigations. Neurosci. Biobehav. Rev. 131, 541–559. doi: 10.1016/j.neubiorev.2021.09.016, PMID: 34606820

[ref47] Spear L. P. (2015). Adolescent alcohol exposure: are there separable vulnerable periods within adolescence? Physiol. Behav. 148, 122–130. doi: 10.1016/j.physbeh.2015.01.027, PMID: 25624108 PMC4484315

[ref48] Spear L. P. (2016). Consequences of adolescent use of alcohol and other drugs: studies using rodent models. Neurosci. Biobehav. Rev. 70, 228–243. doi: 10.1016/j.neubiorev.2016.07.026, PMID: 27484868 PMC5074861

[ref49] Szalardy L. Molnar M. F. Zadori D. Cseh E. K. Veres G. Kovacs G. G.. (2018). Non-motor behavioral alterations of PGC-1α-deficient mice – a peculiar phenotype with slight male preponderance and no apparent progression. Front. Behav. Neurosci. 12:180. doi: 10.3389/fnbeh.2018.00180, PMID: 30210314 PMC6119962

[ref50] Takahashi-Yanaga F. (2013). Activator or inhibitor? GSK-3 as a new drug target. Biochem. Pharmacol. 86, 191–199. doi: 10.1016/j.bcp.2013.04.022 23643839

[ref51] Takahashi-Yanaga F. Shiraishi F. Hirata M. Miwa Y. Morimoto S. Sasaguri T. (2004). Glycogen synthase kinase-3beta is tyrosine-phosphorylated by MEK1 in human skin fibroblasts. Biochem. Biophys. Res. Commun. 316, 411–415. doi: 10.1016/j.bbrc.2004.02.061, PMID: 15020233

[ref52] Tanimizu T. Kenney J. W. Okano E. Kadoma K. Frankland P. W. Kida S. (2017). Functional connectivity of multiple brain regions required for the consolidation of social recognition memory. J. Neurosci. 37, 4103–4116. doi: 10.1523/jneurosci.3451-16.2017, PMID: 28292834 PMC6596582

[ref53] Teixeira-Gomes A. Costa V. M. Feio-Azevedo R. Bastos Mde L. Carvalho F. Capela J. P. (2015). The neurotoxicity of amphetamines during the adolescent period. Int. J. Dev. Neurosci. 41, 44–62. doi: 10.1016/j.ijdevneu.2014.12.001, PMID: 25482046

[ref54] UNODC (2019). World drug report 2019. New York: UNODC.

[ref55] Vogel S. Schwabe L. (2019). Stress, aggression, and the balance of approach and avoidance. Psychoneuroendocrinology 103, 137–146. doi: 10.1016/j.psyneuen.2019.01.020, PMID: 30685681

[ref56] Vorhees C. V. Reed T. M. Morford L. L. Fukumura M. Wood S. L. Brown C. A.. (2005). Periadolescent rats (P41–50) exhibit increased susceptibility to d-methamphetamine-induced long-term spatial and sequential learning deficits compared to juvenile (P21–30 or P31–40) or adult rats (P51–60). Neurotoxicol. Teratol. 27, 117–134. doi: 10.1016/j.ntt.2004.09.005, PMID: 15681126

[ref57] Wang F. He Q. Gao Z. Redington A. N. (2021). Atg5 knockdown induces age-dependent cardiomyopathy which can be rescued by repeated remote ischemic conditioning. Basic Res. Cardiol. 116:47. doi: 10.1007/s00395-021-00888-2, PMID: 34319513 PMC8316897

[ref58] Wang Y. Yin F. Guo H. Zhang J. Yan P. Lai J. (2017). The role of dopamine D1 and D3 receptors in N-methyl-D-aspartate (NMDA)/GlycineB site-regulated complex cognitive behaviors following repeated morphine administration. Int. J. Neuropsychopharmacol. 20, 562–574. doi: 10.1093/ijnp/pyx010, PMID: 28199666 PMC5492807

[ref59] Weaver J. Yang Y. Purvis R. Weatherwax T. Rosen G. M. Liu K. J. (2014). In vivo evidence of methamphetamine induced attenuation of brain tissue oxygenation as measured by EPR oximetry. Toxicol. Appl. Pharmacol. 275, 73–78. doi: 10.1016/j.taap.2013.12.023, PMID: 24412707 PMC3943562

[ref60] Westbrook S. R. Dwyer M. R. Cortes L. R. Gulley J. M. (2020). Extended access self-administration of methamphetamine is associated with age-and sex-dependent differences in drug taking behavior and recognition memory in rats. Behav. Brain Res. 390:112659:112659. doi: 10.1016/j.bbr.2020.112659, PMID: 32437887 PMC7307427

[ref61] Wu J. Zhu D. Zhang J. Li G. Liu Z. Sun J. (2015). Lithium protects against methamphetamine-induced neurotoxicity in PC12 cells via Akt/GSK3beta/mTOR pathway. Biochem. Biophys. Res. Commun. 465, 368–373. doi: 10.1016/j.bbrc.2015.08.005, PMID: 26271595

[ref62] Xiang J. Ran L.-Y. Zeng X.-X. He W.-W. Xu Y. Cao K.. (2021). LiCl attenuates impaired learning and memory of APP/PS1 mice, which in mechanism involves α7 nAChRs and Wnt/β-catenin pathway. J. Cell. Mol. Med. 25, 10698–10710. doi: 10.1111/jcmm.17006, PMID: 34708522 PMC8581309

[ref63] Xing B. Li Y. C. Gao W. J. (2016). GSK3beta hyperactivity during an early critical period impairs prefrontal synaptic plasticity and induces lasting deficits in spine morphology and working memory. Neuropsychopharmacology 41, 3003–3015. doi: 10.1038/npp.2016.110, PMID: 27353310 PMC5101547

[ref64] Xing B. Liang X.-P. Liu P. Zhao Y. Chu Z. Dang Y.-H. (2015). Valproate inhibits methamphetamine induced hyperactivity via glycogen synthase kinase 3β signaling in the nucleus Accumbens Core. PLoS One 10:e0128068. doi: 10.1371/journal.pone.0128068, PMID: 26030405 PMC4452337

[ref65] Xu C. M. Wang J. Wu P. Xue Y. X. Zhu W. L. Li Q. Q.. (2011). Glycogen synthase kinase 3beta in the nucleus accumbens core is critical for methamphetamine-induced behavioral sensitization. J. Neurochem. 118, 126–139. doi: 10.1111/j.1471-4159.2011.07281.x, PMID: 21517846

[ref66] Yan P. Xu D. Ji Y. Yin F. Cui J. Su R.. (2019). LiCl pretreatment ameliorates adolescent methamphetamine exposure-induced long-term alterations in behavior and hippocampal ultrastructure in adulthood in mice. Int. J. Neuropsychopharmacol. 22, 303–316. doi: 10.1093/ijnp/pyz001, PMID: 30649326 PMC6441133

[ref67] Ye T. Pozos H. Phillips T. J. Izquierdo A. (2014). Long-term effects of exposure to methamphetamine in adolescent rats. Drug Alcohol Depend. 138, 17–23. doi: 10.1016/j.drugalcdep.2014.02.021, PMID: 24629630 PMC4066881

[ref68] Zhao R. Chen J. Ren Z. Shen H. Zhen X. (2016). GSK-3β inhibitors reverse cocaine-induced synaptic transmission dysfunction in the nucleus accumbens. Synapse 70, 461–470. doi: 10.1002/syn.21922, PMID: 27377051

[ref69] Zhu H. Yoshimoto T. Imajo-Ohmi S. Dazortsava M. Mathivanan A. Yamashima T. (2012). Why are hippocampal CA1 neurons vulnerable but motor cortex neurons resistant to transient ischemia? J. Neurochem. 120, 574–585. doi: 10.1111/j.1471-4159.2011.07550.x, PMID: 22017466

