# Supplementary figures and images for: Effects of glycogen synthase kinase-3β activity inhibition on cognitive, behavioral, and hippocampal ultrastructural deficits in adulthood associated with adolescent methamphetamine exposure
Source: Front Mol Neurosci. 2023 Mar 6;16:1129553. doi: 10.3389/fnmol.2023.1129553 (PMC10025487; doi:10.3389/fnmol.2023.1129553)

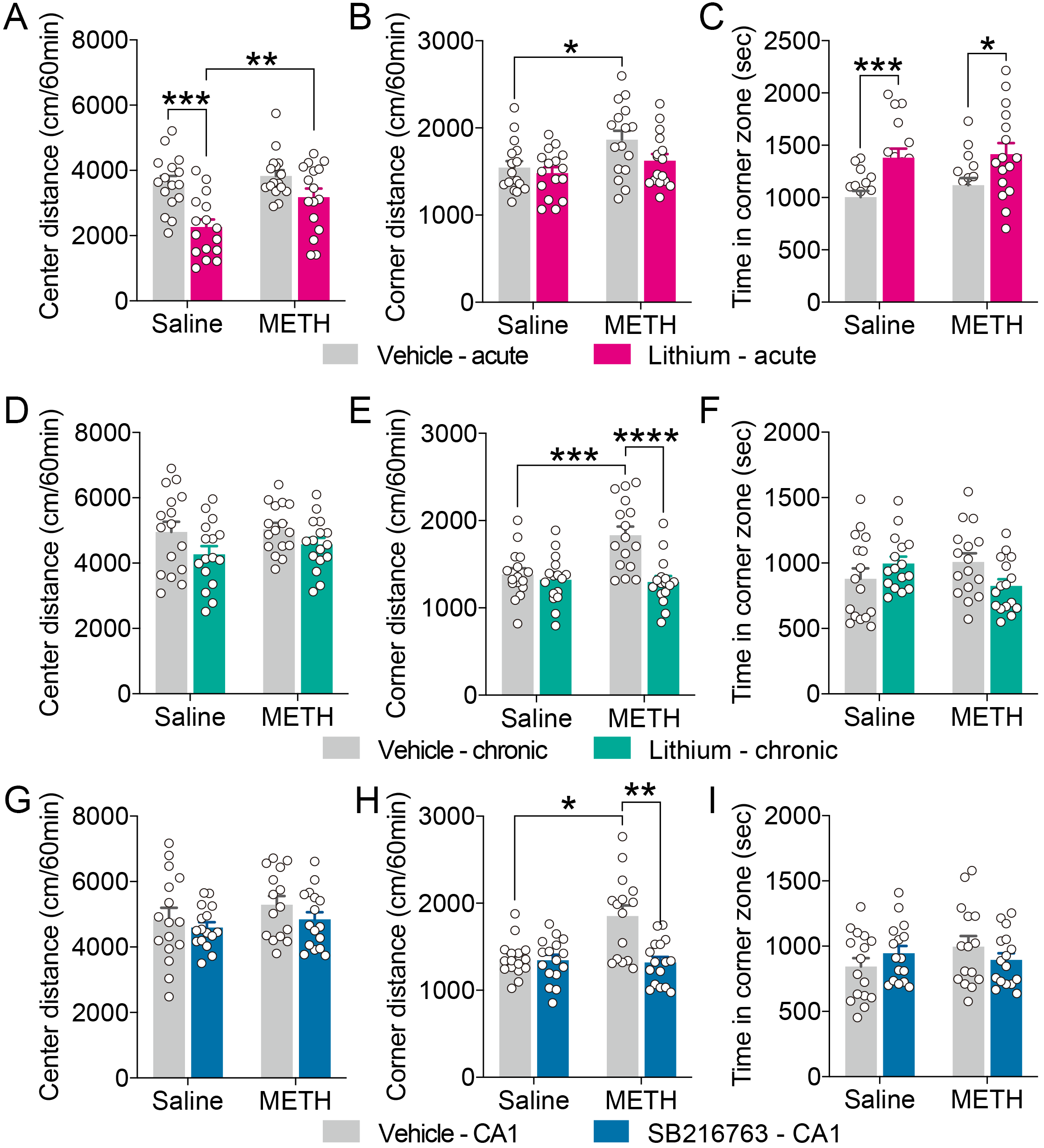

Supplement: Supplementary file 2 [file Image_1.TIF]

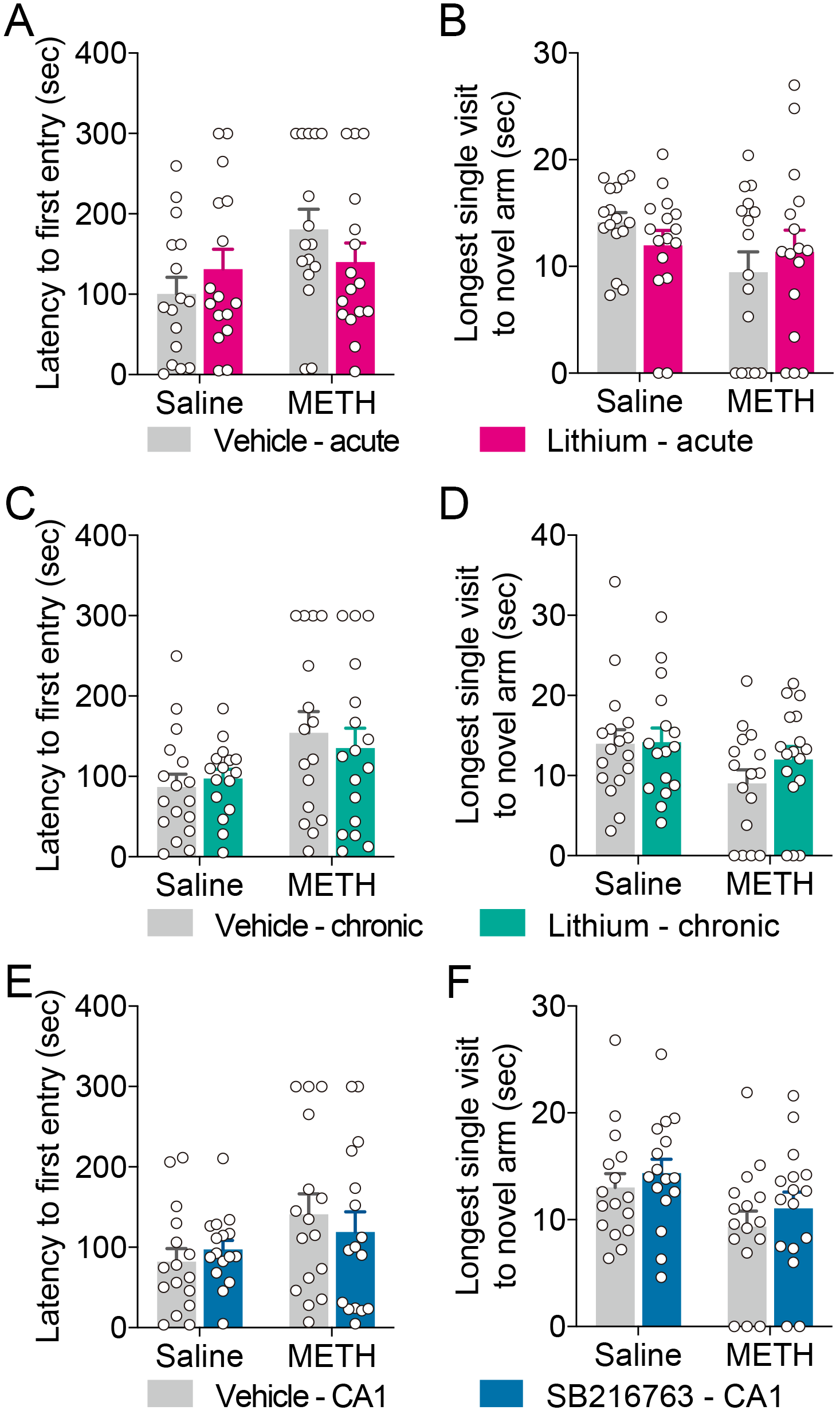

Supplement: Supplementary file 3 [file Image_2.TIF]

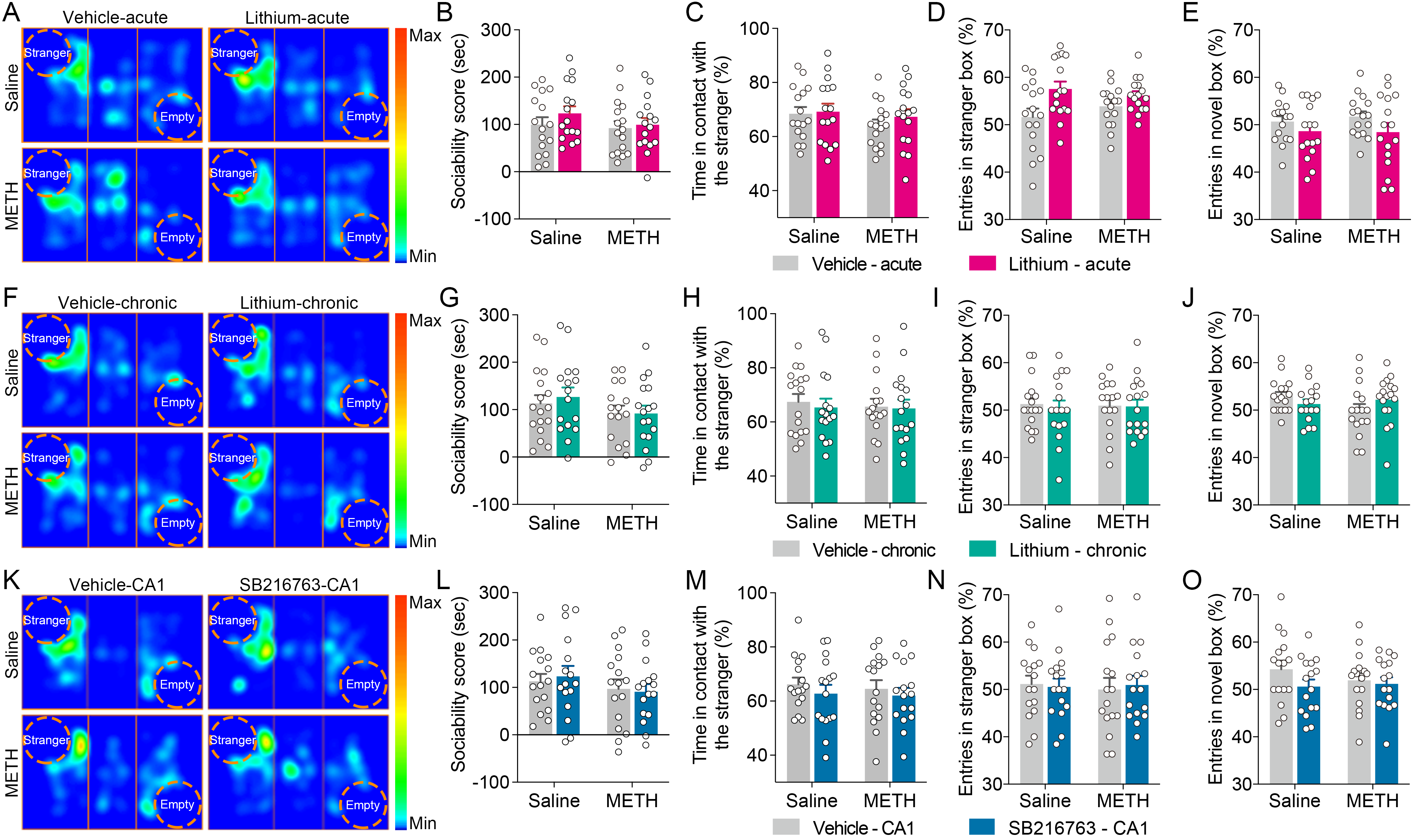

Supplement: Supplementary file 4 [file Image_3.TIF]

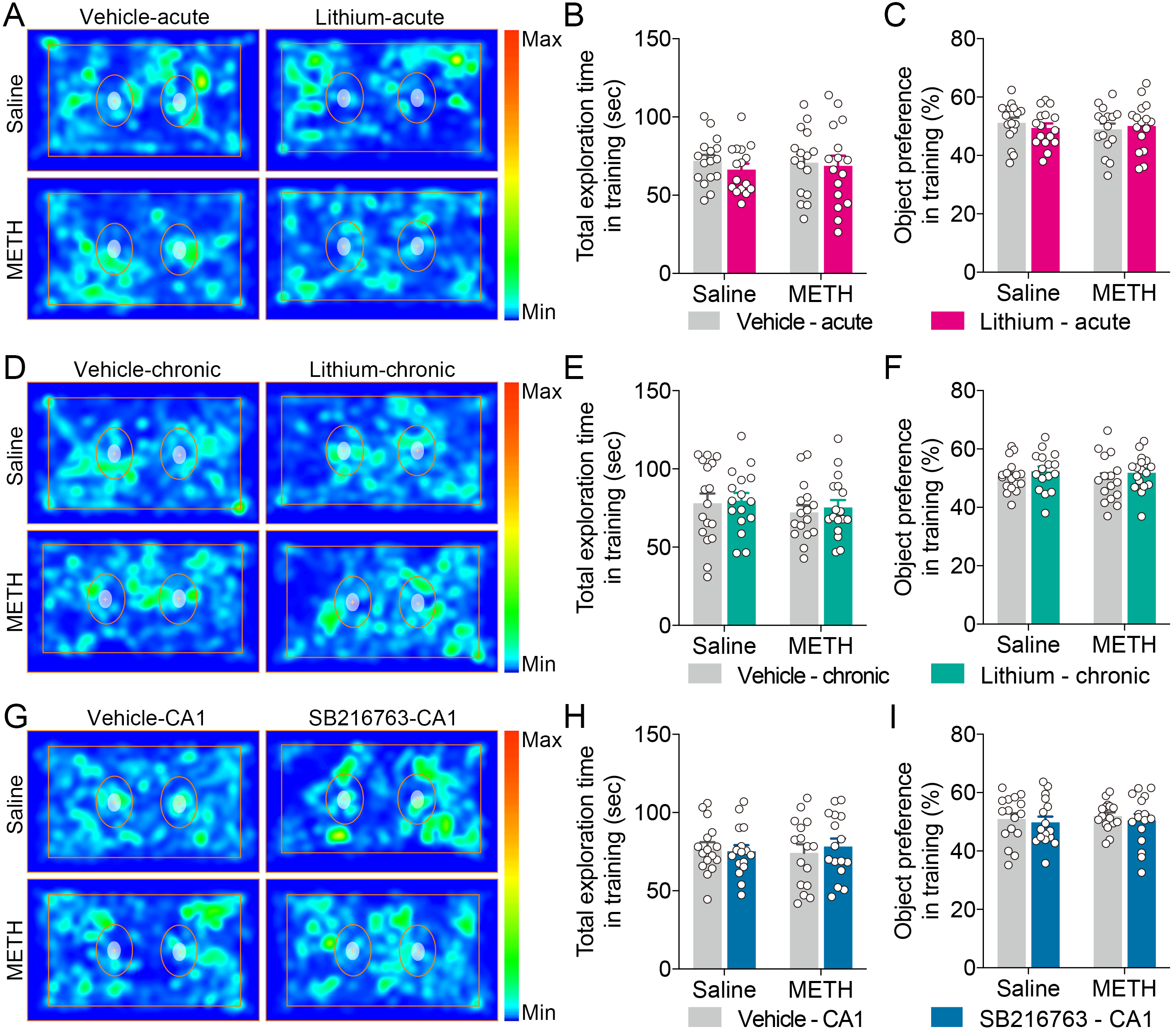

Supplement: Supplementary file 5 [file Image_4.TIF]

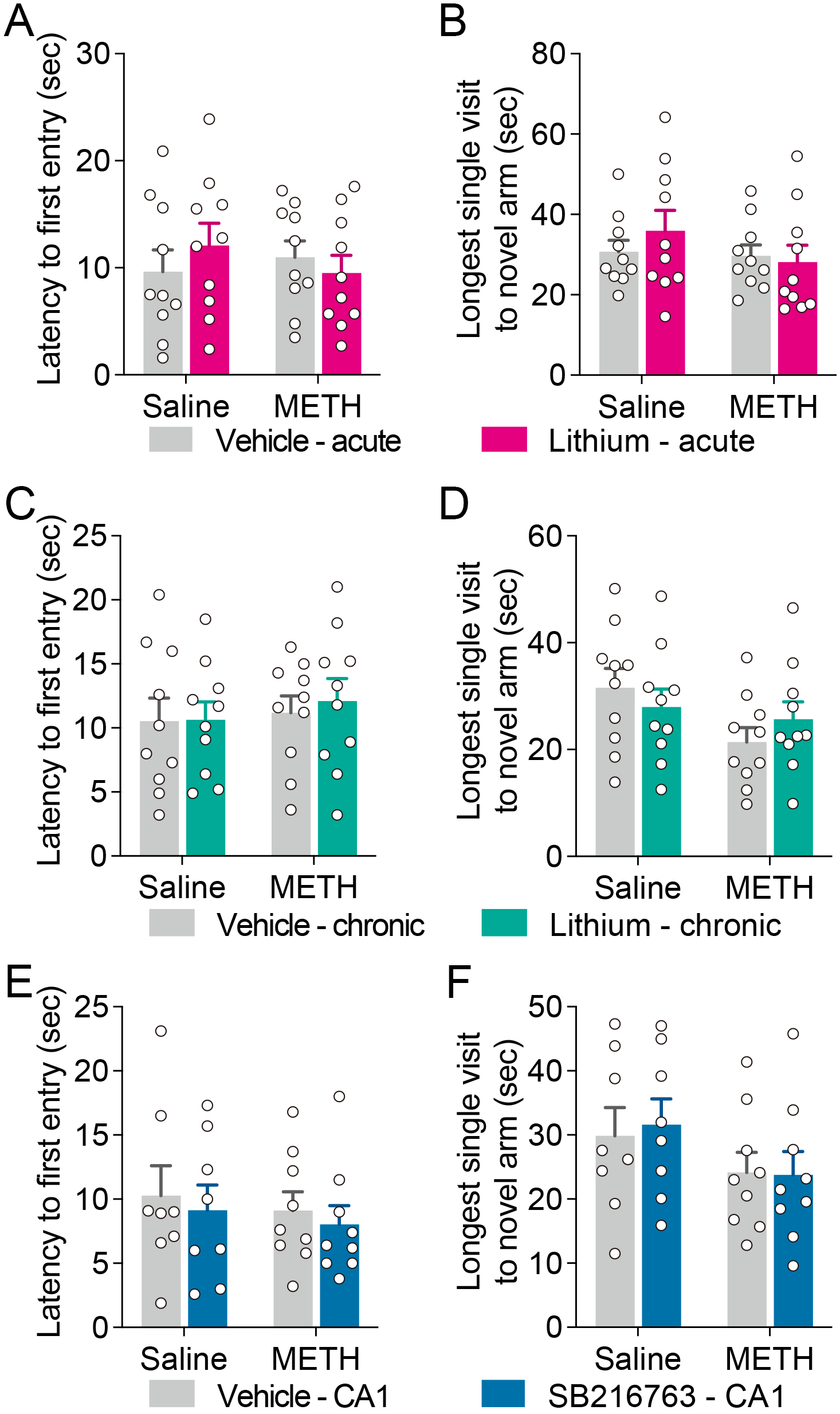

Supplement: Supplementary file 6 [file Image_5.TIF]

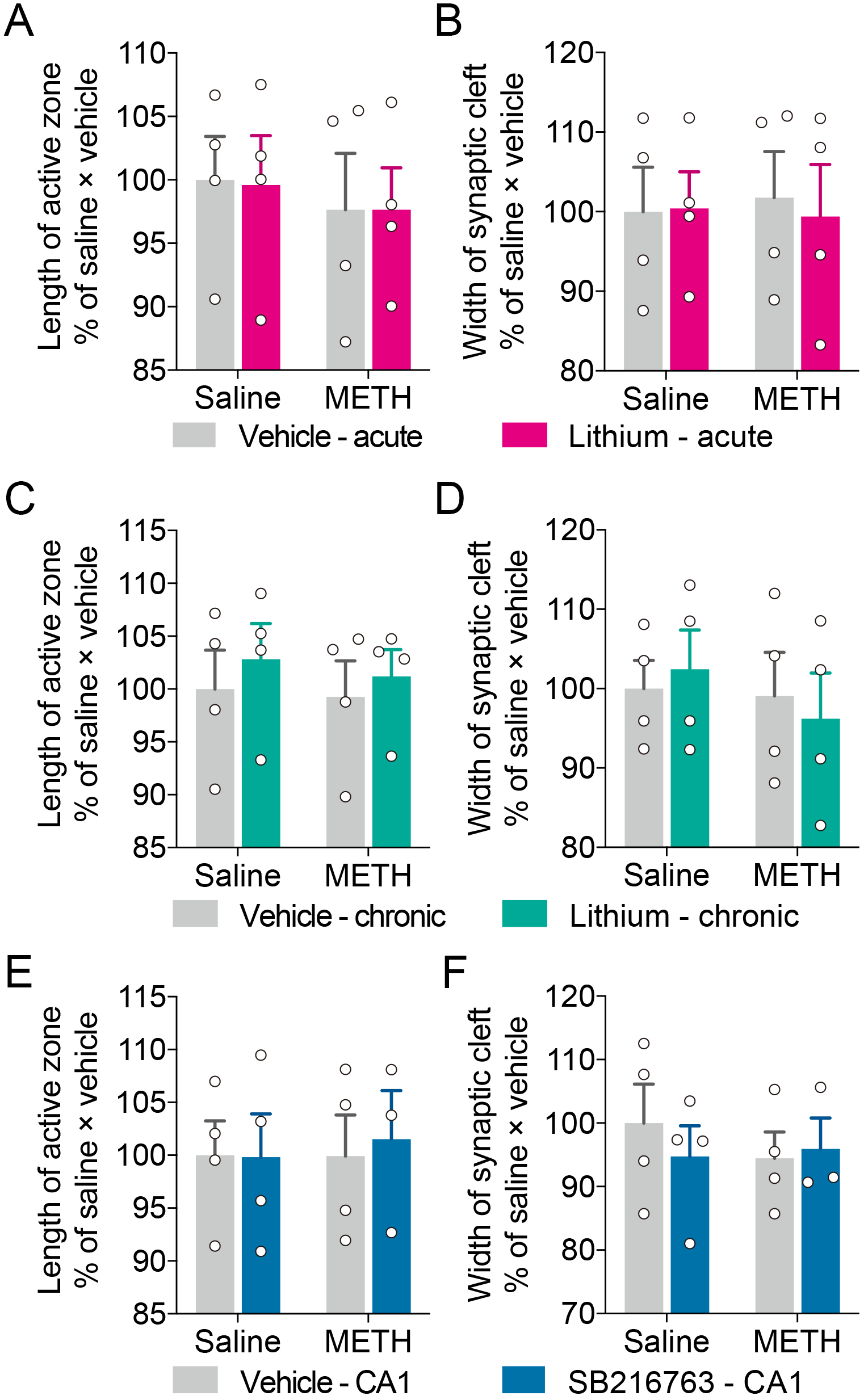

Supplement: Supplementary file 7 [file Image_6.TIF]
